# Pulmonary Delivery of Anticancer Drugs via Lipid-Based Nanocarriers for the Treatment of Lung Cancer: An Update

**DOI:** 10.3390/ph14080725

**Published:** 2021-07-27

**Authors:** Ibrahim M. Abdulbaqi, Reem Abou Assi, Anan Yaghmur, Yusrida Darwis, Noratiqah Mohtar, Thaigarajan Parumasivam, Fadi G. Saqallah, Habibah A. Wahab

**Affiliations:** 1 School of Pharmaceutical Sciences, Universiti Sains Malaysia, Minden, Penang 11800, Malaysia; ibrahimm.abdulbaqi@student.usm.my (I.M.A.); reemabouassi@student.usm.my (R.A.A.); noratiqah@usm.my (N.M.); thaigarp@usm.my (T.P.); fadi_saqallah@student.usm.my (F.G.S.); 2 College of Pharmacy, Al-Kitab University, Altun kupri, Kirkuk 36001, Iraq; 3 Department of Pharmacy, Faculty of Health and Medical Sciences, University of Copenhagen, Universitetsparken 2, DK-2100 Copenhagen Ø, Denmark; anan.yaghmur@sund.ku.dk

**Keywords:** lung cancer, targeted drug delivery, lipid-based nanocarriers, pulmonary delivery, dry powder inhalers, aerosols, liposomes, nanoemulsions, nanotechnology

## Abstract

Lung cancer (LC) is the leading cause of cancer-related deaths, responsible for approximately 18.4% of all cancer mortalities in both sexes combined. The use of systemic therapeutics remains one of the primary treatments for LC. However, the therapeutic efficacy of these agents is limited due to their associated severe adverse effects, systemic toxicity and poor selectivity. In contrast, pulmonary delivery of anticancer drugs can provide many advantages over conventional routes. The inhalation route allows the direct delivery of chemotherapeutic agents to the target LC cells with high local concertation that may enhance the antitumor activity and lead to lower dosing and fewer systemic toxicities. Nevertheless, this route faces by many physiological barriers and technological challenges that may significantly affect the lung deposition, retention, and efficacy of anticancer drugs. The use of lipid-based nanocarriers could potentially overcome these problems owing to their unique characteristics, such as the ability to entrap drugs with various physicochemical properties, and their enhanced permeability and retention (EPR) effect for passive targeting. Besides, they can be functionalized with different targeting moieties for active targeting. This article highlights the physiological, physicochemical, and technological considerations for efficient inhalable anticancer delivery using lipid-based nanocarriers and their cutting-edge role in LC treatment.

## 1. Introduction

Lung cancer (LC) is one of the major medical challenges worldwide. It is globally ranked as one of the most commonly diagnosed cancers, representing about 11.4% of all the reported cases and it is the leading cause for cancer-related deaths, responsible for approximately 18% of all cancer mortalities in both sexes combined [1,2]. In the United States alone, the American Cancer Society predicted that there will be around 235,760 new cases of LC (accounting for 12.4% of all the new diagnosed cancers) and around 131,880 deaths (accounting for 21.7% of all cancer deaths) in 2021. More persons die from LC annually than from cancer of the prostate, breast and colon combined [3,4]. Furthermore, the World Health Organization (WHO) estimates through the Global Cancer Observatory that from 2020 to 2040 the LC incidence and mortality rates for both men and women and all ages will increase by 64.4% and 67.5%, respectively [5,6].

LC may develop as a result of different environmental and genetic factors and their interactions. Tobacco smoking remains the primary cause; smokers are found to have 10- to 30-fold increased risk of developing LC in comparison to non-smokers. Other important factors include second-hand smoke, exposure to industrial and environmental hazards such as radon, asbestos, metals including chromium, cadmium and arsenic, exposure to different organic chemicals, ionizing radiation and a positive history of respiratory illnesses (e.g., bronchitis, emphysema, and tuberculosis). In families, first-degree relatives of LC probands have a 2- to 3-fold increased risk of LC and other malignancies, many of which are not smoking-related [7].

LC is classified into two main types, small cell lung cancer (SCLC) and non-small cell lung cancer (NSCLC). The latter is subdivided depending on the tumor tissue histology into three main histologic categories, including adenocarcinoma, squamous cell carcinoma, and large-cell carcinoma. NSCLC represents approximately 85% of all lung cancers, while SCLC is responsible for the remaining percentage [7,8]. The detection of LC at its early stages is crucial for best therapy outcomes, but unfortunately, the symptoms typically start to appear only at the advanced stages of the disease and sometimes they are masked by other concurrent respiratory conditions. Accordingly, the majority of patients are diagnosed with LC while the disease is at its advanced stages and turned out to be incurable with currently available treatments [9] with very poor prognosis and a 5-year survival rates of only 21% [4].

The treatment strategy depends on the type, stage of LC and the physical state of the patient. The currently available conventional treatment methods may include surgery, high doses of intravenous chemotherapeutic agents, radiation therapy, targeted therapies, immunotherapy, and photodynamic or laser therapy [7,10]. Generally, surgery is confined to the early stages of LC and is typically combined with chemotherapy and/or radiation therapy to eradicate the cancerous tissue [9,11]. The use of single chemotherapeutic agents (such as cisplatin, paclitaxel, and etoposide) or their combinations remain the main treatment method for LC. However, the therapeutic efficacies of these cytotoxic drugs are limited due to their poor selectivity, the development of multidrug resistance, and besides, their use is associated with severe adverse effects and systemic toxicity symptoms including anemia, nausea, vomiting, nephrotoxicity and neurotoxicity which in turn limit their use [12,13]. Therefore, and for a complete cure and eradication of LC, there is an immediate need to use and investigate the possible potential roles of different routes of drug administration such as the pulmonary route and novel drug delivery systems such as nanoscale materials that are highly effective with excellent targeting abilities against the LC cells and display improved toxicity profiles.

Nanotechnology represents a powerful tool in the hands of researchers today for enhancing the currently available classical therapies and developing new therapeutic strategies and diagnostic tools to combat LC. The extensive research in this field has yielded a wide range of nanosystems (including the lipid-based nanocarriers) that have the potential to dramatically change how LC is treated nowadays [13,14,15]. This is attributed to their ability to entrap drugs with different physicochemical properties, suitability for combination therapy, and enhanced permeability and retention (EPR) effect which makes them highly effective in passive targeting, besides; their surface could be functionalized with different targeting moieties for active targeting, so they can selectively target cancerous cells and neoplasms.

In this contribution, the potential role, advantages, and challenges associated with using the pulmonary route to deliver anticancer drugs via lipid-based nanocarriers are presented. The physicochemical aspects that should be considered for efficient delivery, the recent technologies, materials, and lung delivery devices used to formulate and deliver different anticancer drug-loaded lipid-based nanocarriers are discussed. Furthermore, the advances in using the inhalable lipid-based nanocarriers for combating LC and their evaluation on the in vitro, in vivo, and clinical studies are presented.

## 2. Methodology

The literature selection in this review was performed by manually searching the PubMed, Google Scholar, ScienceDirect, and Wiley Library databases for published literature on inhalable chemotherapy via lipid-based nanocarriers using various keywords such as (Inhaled/aerosolized/nebulized/dry powder inhalers/inhalable chemotherapy for LC, inhaled liposomes for LC, aerosolized solid lipid nanoparticles (SLNs) for LC, DPIs of nanostructured lipid carriers (NLCs) for LC, inhalable nanoemulsions (NEs) for LC, lipid-based nanoparticles for LC, etc.). For liposomes, examples of the most recent (2010–2021) studies about inhalable anticancer drug-loaded liposomal formulations that involved in vivo studies were included in this study. All the published research work for the other types of lipid-based nanocarriers (i.e., NEs, SLNs, NLCs, niosomes, and others) designed as inhalable anticancer drug-loaded formulations for the treatment and/or diagnosis of LC were reviewed in this study.

## 3. Inhalable Anticancer Therapy via Lipid-Based Nanocarriers: Main Advantages and Critical Challenges

Drug delivery for the treatment of LC using lipid-based nanocarriers is achieved mainly via the intravenous and pulmonary routes of administration. Regional drug delivery methods at the tumor site are also considered for certain cases. 

Pulmonary delivery of anticancer drugs via lipid-based nanoparticles for LC treatment is a growing and expanding area of research. This route of drug administration is non-invasive (needle free), provides better patient compliance, and can be self-administered. It can be used to overcome the drawbacks associated with the oral or intravenous routes that may include high levels of systemic toxicity, poor aqueous solubility of the anticancer agents, low drug accumulation within the tumor, and high rates of tumor relapse [16].

The use of inhalable lipid-based nanocarriers could provide many advantages over the conventional routes for LC treatment, especially for patients with surgically unresectable LC. Pharmacokinetically, inhalation allows the delivery of chemotherapeutic agents to the target cancer cells and avoids the hepatic metabolism; thus, rapid onset of action, lower dosing and fewer systemic distribution and toxicities are expected [17]. Moreover, the alveolar region in the lungs has a large surface area of ~100 m^2^, extensive vasculature, and limited drug-metabolizing enzymatic activity compared to other organs such as the liver and the gastrointestinal tract. In addition, the alveolar epithelium is extremely thin (0.1–0.2 μm), which is much thinner than that in the upper bronchial tree (50–60 μm). Thus, drug absorption and bioavailability in the targeted region may be further improved [18,19]. Additionally, phospholipids, which are major constituents of many lipid-based nanoparticles, especially liposomes, are present in the lungs and constitute almost 90% of lung surfactants [20]. This favors the design of more biocompatible lipid-based formulation and enhances lung tolerability to the delivered anticancer agent. All these factors may significantly decrease treatment failures, development of drug resistance, and chemotherapy interruptions that are responsible for the repopulation of cancerous cells. Subsequently, tumors refractory to traditional systemic chemotherapy could also potentially respond to inhalational therapy.

**Figure 1:**
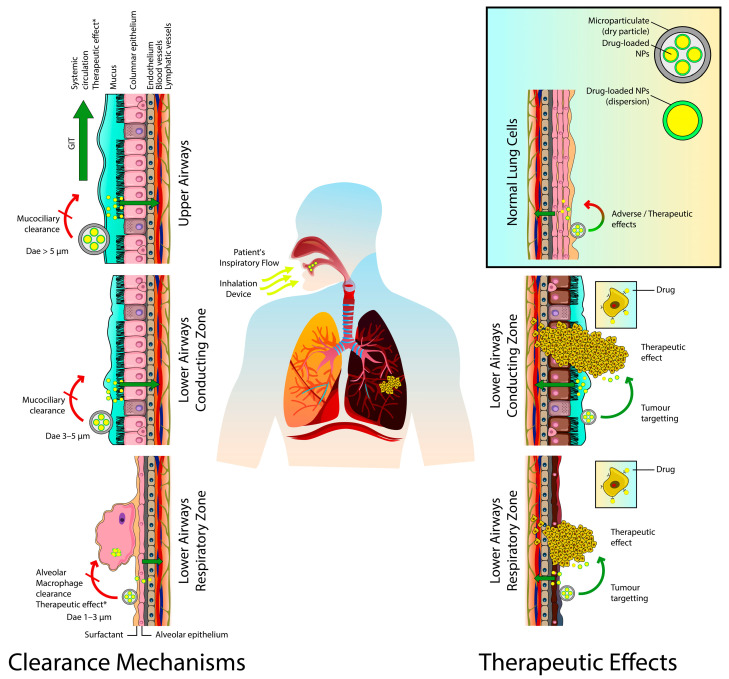
The involved clearance mechanisms and therapeutic effects of the inhaled anticancer agents-loaded nanocarriers. D_ae_, aerodynamic diameter; GIT, gastrointestinal tract; NPs, nanoparticles. * Therapeutic effects are obtained via expression/secretion of anticancer proteins or induction of anticancer immune responses.

Pulmonary drug delivery via lipid-based carriers allows anticancer drugs to target and reach various lung tumors via different pathways. After deposition in the respiratory tract, the inhaled drug can target lung tumors by directly penetrating the tumor via the achievement of elevated local concentrations and significantly high concentration gradients of therapeutic agents at the lung tumor site. Certain types of lung tumors such as squamous cell carcinomas or bronchioloalveolar cell carcinomas that are found next to or within the airways might take up the deposited drug by direct penetration. Furthermore, drugs delivered to the lung by inhalable lipid carriers can be absorbed into the local blood circulations. Due to the communication between the bronchial and pulmonary circulations, sufficient drug concentration could reach lung tumors that lack a direct connection with the main airways depending on the tumor site. The bronchial vasculature nourishes lung tumors if they are located in the conducting region, while the pulmonary circulation feeds them if they are sited at the respiratory region [21,22,23]. However, the absorption, lung clearance mechanisms, biodistribution, and tumor penetration of inhaled drug-loaded particles are subject to many factors such as the physicochemical properties of the drug/particles, the characteristics and composition of the used formulation, the site of dug deposition, the histological features of the respiratory system, the associated pathological condition. In this regard, Haque et al. evaluated how inhaled liposomal formulation affects existing lung disease by comparing the pulmonary pharmacokinetic behavior of drug-loaded ^3^H-labelled PEGylated liposomes after intratracheal administration to healthy rats and rats with bleomycin-induced lung inflammation by following both ^3^H label and drug. The results showed that liposomes were initially cleared more rapidly from inflamed lungs than from the healthy ones but exhibited similar rates of lung clearance after three days. This was interesting given that mucociliary clearance was more efficient from healthy lungs, despite evidence of higher mucus retention in inflamed lungs and reduced association of the liposomes with lung tissue. The plasma pharmacokinetics of ^3^H-phosphatidylcholine revealed higher liposomal bioavailability and more prolonged absorption from inflamed lungs. Concentrations of the pro-inflammatory cytokine IL-1β were increased in bronchoalveolar lavage fluid after a single pulmonary dose of liposomes to rats with inflamed lungs, but no other significant changes in inflammatory lung markers were identified in healthy or bleomycin-challenged rats [24]. Moreover, inhaled drugs are also drained by the lymphatic system; they were commonly detected in the lungs’ lymph nodes (Figure 1). Consequently, these nodes are considered as potential targets for the inhaled drug to suppress cancer metastasis to and from the lungs [25,26,27]. Videira et al. described the biodistribution of radiolabeled (^99m^Tc) solid lipid nanoparticles (SLNs) following their pulmonary delivery to male Wistar rats. A 60 min dynamic image acquisition was performed in a gamma-camera, followed by static image collection at 30 min intervals up to 4 h post inhalation. Radiation counting was performed in organ samples collected after the animals were sacrificed. The results revealed a significant uptake of the radiolabeled SLNs into the lymphatics after inhalation and a high distribution rate in periaortic, axillar, and inguinal lymph nodes [28]. Due to all these remarkable advantages inhalation therapy is having the potential to become an effective and safe delivery method for the treatment of LC.

Despite the aforementioned advantages, we should bear in mind that the pulmonary drug delivery of anticancer drugs via lipid-based nanocarriers for LC treatment is confronted by some challenges and limitations. One of the major concerns is the lung tolerance and the potential risk of local pulmonary toxicity and adverse effects because of the cytotoxic activity of anticancer drugs themselves. Besides, the lungs’ health of LC patients is often impaired either due to LC complications or because of the presence of other concomitant lung diseases such as asthma or chronic obstructive pulmonary diseases that can significantly affect patients’ ability to tolerate the inhalable anticancer therapy.

Results from the so far conducted and published clinical trials (see Section 7) of nebulized liposomal chemotherapeutics such as 9-nitrocamptothecin (9NC) (phase I) [29], cisplatin (CIS) (phase I and Ib/IIa), (NCT00102531) [30,31,32] for the treatment of LC revealed that they have relatively safe profiles. The most reported side effects or dose-limiting toxicities (DLT) were mainly related to the respiratory tract, where grade 3 chemical pharyngitis and grade 3 bronchitis are reported as the most severe side effects [29,30]. To reduce these adverse effects, prophylactic doses of bronchodilators and/or corticosteroids before starting the anticancer inhalation therapy were used and/or recommended in clinical trials; they were found to help controlling these effects [29,33,34,35]. 

Furthermore, the inhaled drug-loaded particles are faced by various lung clearance mechanisms depending on various factors (Figure 1). These mechanisms can clear these particles from the lungs before reaching their targeted sites or reduce their residence time before exerting their desired therapeutic effects. The mucociliary clearance is the predominant mechanism in the conducting zone; the inhaled particles will be carried from the bronchial region to the larynx and then transferred to the gastrointestinal tract by swallowing. Almost 80 to 90 percent of the inhaled particulates can be excreted from the central and upper airways by this mechanism within 24 h. The lining mucus blanket (with thickness up to 30 μm) secreted by the goblet cells in this region represents another barrier. Additionally, particles on the alveolar epithelium (respiratory zone) may be phagocytosed by alveolar macrophages leading to lysosomal degradation, or they are taken to the upper respiratory tract by mucociliary escalator. The macrophages tend to engulf particles with geometric size of 0.5 to 5 μm. The alveolar epithelium, on the other hand, is covered by lung surfactants which can aid in drug dissolution and diffusion. If drugs are dissolved, they are either absorbed by the blood or lymphatic circulations or subjected to enzymatic degradation [36,37,38,39,40]. Lipid-based nanocarriers were employed efficiently to overcome these challenges and obtain improved therapeutic outcomes by ensuring longer drug-residence time and sustained release of therapeutic agents in the targeted sites of the lungs. Xu et al. developed a spray-dried liposomal formulation of vincristine and tested the absorption and tissue distribution of the drug after the intratracheal administration of the formulation in male SD rats. The liposomal formulation was able to enhance the pharmacokinetic behavior of the drug by decreasing drug clearance and elimination half-life by 83.2% and 81.1%, respectively, compared to the free drug solution [41]. After pulmonary delivery of paclitaxel-loaded, surface-modified solid lipid nanoparticles (SLNs) with a folate-grafted copolymer of polyethylene glycol (PEG) and chitosan, the formulation was found to significantly prolong the pulmonary exposure to the drug to up to 6 h in vivo (in female CD-1 and BALB/c mice) and limit the systemic distribution of the drug compared to inhaled Taxol-like formulation [42].

## 4. Physicochemical Considerations, Passive, and Active Targeting For Efficient Pulmonary Delivery of Anticancer Drugs via Lipid-Based Nanocarriers

The physicochemical properties of the anticancer drugs, nano or microcarriers, should be well considered while designing inhalation formulations because they will affect the drug residence time within the lungs and, consequently, the therapeutic efficacy. In order to achieve the required pharmacodynamic effects, anticancer agents must be available to cancerous cells within a minimum period of time. Drugs that are readily absorbed by the lungs may then be ineffective in treating the disease. Generally, lipophilic drugs with (log P > 0) are absorbed rapidly from the lungs because of their higher ability to diffuse in the lipid membranes. In contrast, hydrophilic drugs (log P < 0) tend to have longer lung residence times [43]. As a result, formulation techniques to increase lung residence of the lipophilic drugs and prolong their exposure time to cancer must be adopted.

Aerosols of drug-loaded lipid-based nanoparticles (either as liquid dispersions or dry powders) can be deposited via various mechanisms such as inertial impaction, sedimentation, and Brownian diffusion on the respiratory epithelium. These mechanisms are governed primarily by the aerodynamic diameter (D_ae_) of the generated aerosol particles. The D_ae_ is the most precise parameter for measuring the aerosol particle size. It can correlate the particles’ dynamic behavior as it is calculated based on their geometric size, density, and dynamic shape. There is a consensus in the literature that for efficient pulmonary delivery, the inhaled particles should be with D_ae_ of (1–5 µm) to reach the lower respiratory tract and in the range of (1–3 µm) for the respiratory zone. Particles with (D_ae_ > 5) µm will be deposited in the upper respiratory tract, while smaller particles those with (D_ae_ < 0.5) µm are expected to be emitted out of the body via the expiratory airflow [44,45,46,47]. The lipid-based nanocarriers, can be aerosolized and delivered to the lungs as dispersions by nebulization. At the same time, due to their extremely small geometric size, they must be incorporated in secondary carriers (microparticles) to be delivered as dry powders. Various particle engineering techniques were applied for their preparation and are discussed in Section 5.

Particle shape can also substantially contribute to the developed inhaled anticancer formulation’s therapeutic efficiency because it can determine the extent of alveolar macrophage clearance. The relationship between different particles’ shapes (i.e., elliptical disks, spherical, oblate ellipsoids, rectangular disks, and worm-like shape) and the time that was taken for their clearance by phagocytosis was previously investigated [48,49]. It was found that the shape and orientation of these particles significantly affect their phagocytosis clearance time. Phagocytosis was initiated for all shapes in at least one orientation. Due to macrophages’ attachment to their principal axes, elliptical discs were engulfed in less than 6 min. Regardless of the macrophage attachment point, the spherical particles were also cleared immediately. Interestingly, macrophage attachment to the flat surfaces or minor axes of the rectangular, elliptical disks, and oblate ellipsoids failed to clear these particles even after two h [48]. Furthermore, because of their low curvature region, worm-like shaped particles resulted in significantly less phagocytosis clearance than the spherical particles [49]. By the use of particle-engineering technologies, lipid based nanocarriers could be embedded in microparticles of different morphologies for possibly enhancing their delivery.

The surface charges of the inhaled particles could also influence their cellular uptake in addition to their clearance and retention in the lungs. The positively charged particles were reported to have better penetration into tumor cells because of their higher binding tendency with tumor cells [36]. Furthermore, cationic nanoparticles were shown to be taken up quickly by the lung epithelial cells and or macrophages shortly after their administration, unlike neutral and anionic nanoparticles of the same hydrodynamic diameters. Therefore, cationic nanoparticles are retained for a longer time within lung cells, limiting their translocation to lymph nodes and bloodstream [50]. 

Targeting cancerous cells via drug-loaded lipid-based nanoparticles could be done by the passive and active methods. Generally, via passive targeting, nanoparticles tend to leak preferentially into cancer tissue via permeable tumor vessels and are then retained in the tumor bed due to reduced lymphatic drainage. This phenomenon is known as the enhanced permeability and retention (EPR) effect [51]. However, the EPR effect is suggested to offer less than a twofold increment in drug delivered by a nanocarrier to tumors in comparison to critical healthy organs, resulting in subtherapeutic concentrations that are not sufficient to cure most cancers [52]. On the other hand, the active targeting can enhance the therapeutic efficacy and increase the selectivity of drug delivery by attaching targeting ligands (which bind to specific receptors on the tumor cells and endothelium) to the surfaces of nanocarriers [53]. The surfaces of the lipid-based nanocarriers are highly tunable and could be functionalized using with more than one type of functional groups and surface modification techniques to provide stealth characteristics (PEGylation), and active targeting towards the cancerous cells [54,55]. The main targeting sites in LC may include the overexpressed receptors on the surfaces of the cancer cells (e.g., epidermal growth factor receptor (EGFR), folate receptors (FRs), transferrin receptors (TfRs)), cellular organelles (e.g., mitochondria, lysosomes) and the LC microenvironment (e.g., vascular cell-adhesion molecules, cluster-of-differentiation 44, matrix-metalloproteases) [56]. The strategies of developing positively charged or surface modified uni, di, or multifunctional lipid-based nanocarriers using different ligands and targeting moieties for active targeting of LC are adopted by many researchers. The results of these studies in enhancing the pulmonary delivery and therapeutic efficiency of anticancer drugs on the in vitro and in vivo levels are discussed and summarized in Section 6 of this article. Another active targeting method of malignant cells can be achieved by the development of “stimuli-responsive” nanocarriers by taking advantage of the natural physiological conditions within the target tissue, such as elevated temperature or alteration in pH, or through the application of external stimuli such as a magnetic field or ultrasonic waves [54]. However, the potential role of different types of inhaled stimuli-responsive lipid-based nanoparticles, such as the thermo-sensitive, pH-sensitive, magnetic-field, and ultrasound responsive nanocarriers in the treatments of LC, is rarely investigated. In one study, inhaled magnetic and thermo-responsive lipid vehicles were incorporated with superparamagnetic iron-oxide nanoparticles and budesonide for controlled and targeted pulmonary delivery. The formulated dry powders had a fine particle fraction (FPF) of 30%. The formulations were shown to have an accelerated drug release rate at hyperthermic temperatures (45 °C). The authors concluded that the developed lipid matrix is a good and effective drug vehicle in targeted and controlled inhalation therapy [57].

In addition to the discussed physicochemical aspects, the pathophysiological aspects of the lungs should also be considered while developing an inhalable formulation for the treatment of LC. These might include LC type and stage, concomitant diseases such as asthma, chronic obstructive pulmonary diseases, and their associated changes to normal lung physiology should also be considered while developing an inhaled formulation for the treatment of LC. These considerations are well-reviewed and discussed elsewhere [36,58].

## 5. Devices for the Pulmonary Drug Delivery of Anticancer Drug-Loaded Lipid-Based Nanocarriers

Drug-loaded lipid-based nanoformulations are delivered to lungs as liquid-based (i.e., solutions, dipersions) or solid-based (i.e., dry powders) aerosol systems. Nebulizers, dry powder inhalers (DPIs), pressurized metered-dose inhalers (pMDIs), and soft-mist inhalers are the main types of devices to deliver therapeutic agents into the lungs.

For effective therapeutic outcomes, higher doses (ranging from one to tens of mg) of inhaled anticancer drugs must be deposited in the lungs. The pMDIs and soft-mist inhalers can only deliver smaller drug doses of less than 1 mg; therefore, they are rarely used to deliver anticancer drugs [59,60]. On the other side, nebulizers and DPIs are suitable for delivering higher drug doses necessary for the inhaled chemotherapeutic agents to act on the cancerous cells and tumors. Therefore, these devices have the potential to be used effectively for inhalable-based anticancer therapy.

Nebulizers are liquid-based aerosol delivery devices. Different types of these devices are available, including jet, vibrating mesh, and ultrasonic nebulizers; they deliver aerosols to the lungs as finely atomized droplets with high FPF over certain periods of time using compressed gas flow, oscillating perforated membrane, or piezoelectric crystals vibrating at high frequency, respectively [61]. They are the most used delivery systems of anticancer drugs-loaded lipid-based nanoparticles in preclinical studies and the only used ones in pilot studies and clinical trials (see Section 7). Nebulizers have many potential benefits. They generate large amounts of aerosolized droplets with an aerodynamic size of <5 μm from solutions or nanoparticles dispersions to be deposited in the lungs. Also, they need minor patient collaboration and are suitable for patients with chronic pulmonary illnesses such as LC who cannot perform active inhalation or receiving mechanical ventilation [62]. However, for the delivery of therapeutic doses, the nebulization process may need to continue over a long period and for multiple cycles. Furthermore, during nebulization, large amounts of the produced aerosols are not inhaled but instead, they are lost in the nebulizer or released into the air leading to air contamination. Therefore, the nebulization of anticancer drugs needs to be performed under hospital settings only, as specific protective and safety measures should be taken to protect healthcare givers and neighbors and prevent their exposure to chemotherapeutic agents. On the other hand, factors such as pH, osmolality, and viscosity of the developed inhaled nanoformulations dispersions should be well characterized and optimized for efficient delivery and prevent coughing, lung mucosa irritation, and bronchoconstriction [27,63,64]. Furthermore, the nebulization process of lipid-based nanoparticles using the different types of nebulizers could significantly affect the size, drug loading, and the in vitro release rate of these carriers. It was reported that during nebulization by jet nebulizer, the multilamellar liposomes (with particle size (PS) of up to several microns) exhibited a decrease in PS, while unilamellar liposomes (with PS from 30 to 150 nm) have shown an increase in PS [65]. By testing the nebulization of paclitaxel-loaded lipid nanocapsules using jet, ultrasonic, and mesh nebulizers of different brands, it was revealed that vibrating mesh nebulizers were able to generate aerosols of lipid nanocapsules with good performance and stability [66]. The excipients of nanoparticles could also contribute to the stabilization of nanoparticles structure during the nebulization process. It was reported that the incorporation of cholesterol and PEGylated phospholipids in the liposomal formulations could result in an increase in liposome membrane stability in the broncho-alveolar lavage or during nebulization [67,68]. Therefore, the proper selection of nebulization technique and formulation excipients should be very well considered while developing lipid-based nanocarriers for the LC via nebulization. They could significantly contribute to the nanoparticle stability and consequently the therapeutic outcome of the developed formulation.

On the other hand, the solid-based delivery aerosol systems (i.e., DPIs) can overcome the previously mentioned drawbacks of nebulizers as they have many advantages and unique features [69]. They are easy to use, can be self-administered, portable, do not need hospitalization, cost-effective, and can efficiently deliver high doses of anticancer drugs or drug-loaded nanocarriers as dry powder to the lungs [70]. DPIs are breath-actuated using the patient’s inspiration for a short time with negligible drug exhalation, causing no air contamination during use. Furthermore, the dry powders have higher long-term stability, suitable for the formulation of lipophilic drugs [71]. Besides, DPIs can be produced as disposable devices, consequently limiting the contamination of the device and the environment [72]. Recently, many preclinical studies were published, including the development of inhaled anticancer drugs using DPIs for the treatment of LC, which reflects the growing interest in this approach [73,74,75]. However, the development of dry powders for such drugs for DPIs necessitates the need for taking extra protective and safety measures by the researchers and personnel in the industrial facilities if the formulation is to be commercialized.

Nowadays, there is a wide range of the available classical DPIs in the markets, and the number will keep increasing. The main differences among these DPIs lie in their design, airflow resistances, formulations’ type and excipients, and dry powder production techniques and dispersion methods [76,77]. These mentioned device and formulations related-variables, in addition to the patient-related variations such as the patient’s respiratory health and performance, may significantly affect the performance of these devices and lead to some variations in their drug deposition efficiencies into the lungs [78].

To ensure efficient powder aerosolization and delivery of drugs, the production of classical DPIs needs many optimization steps where the milled and micronized drug particles are usually formulated as three main particle types, namely: carrier-based, agglomerate-based (spheronized), and engineered particles. In the carrier-based type, the drug particles are attached physically to large inactive carrier particles such as lactose (if lactose was the used carrier they are called as lactose blends), while the agglomerates are composed of aggregates of the micronized drug. The engineered particles are usually composed of spray-dried particles of drug solubilized in an inert hydrophobic carrier [79].

On the other hand, nanocarriers-based DPIs also require many steps to create the inhalable drug-loaded nanocarriers dry powder beside the initial preparation and optimization of the drug-loaded nanocarriers processes. As discussed previously, the inhaled particles’ D_ae_ must be in the range of (1–5 µm). Since the lipid-based nanoparticles possess too small D_ae_ (due to their small particle size and or density) so they are not suitable by themselves for efficient deposition in the respiratory tract, where they may be exhaled out of the respiratory system. Besides, lipid-based nanoparticles’ high surface free energy due to their small size and enormous surface area can lead to particle aggregation, making their handling as a dry powder very difficult because of the poor flowability [44,80]. Overcoming these limitations of these nanoparticles can be done by particle engineering. One of the available potential solutions is to embed nanocarriers into microstructures (microparticles) with the required aerodynamic properties [81,82,83,84]. These nano in microparticles are also known as nanoaggregates or Trojan particles [85,86]. They must be engineered to have good dispersion properties to quickly dissolve and redisperse to release the initial nanocarriers in lung fluids upon delivery. The lipid-based nanocarriers, could be encapsulated into these microscale structures.

The excipients used in the formulation of dry powders of the nano in microparticles are typically hydrophilic excipients such as lactose, trehalose, dextran, and mannitol [87,88]. However, other additional materials were investigated such as L-leucine [89,90], hydroxypropyl β -cyclodextrin, polyvinyl alcohol, whey protein, maltodextrin, and gum Arabic [91].

Different techniques were used to produce dried lipid-based nanoparticles with or without excipients to generate stable, well-characterized, and inhalable particulates. These include spray-drying, freeze-drying (lyophilization), spray freeze-drying, milling, supercritical fluid drying, and electrohydrodynamic (electrospraying and electrospinning) methods. The pros and cons of these techniques, the critical variables that should be considered during formulation, and the properties of dry powders produced are well discussed and reviewed elsewhere [92,93].

Effervescent technology was also used to overcome the lipid-based nanoparticles’ size-related limitations and enhance their lungs’ release. It is done by embedding and co-drying of nanoparticles with an effervescent matrix, the typical excipients used in effervescent-based dry powders may include sodium carbonate, citric acid, and ammonium hydroxide.

The concept was first introduced by Ely et al. 2007 for polymer-based nanoparticles using ciprofloxacin as a drug model [94]. The technology was applied later to develop inhalational dry powders of cytotoxic drug-loaded lipid [95] or polymer-based nanoparticles [96,97] to treat LC. In one study, a comparison between inhalable effervescent-based and non-effervescent nanostructured lipid particles of 9-Bromo-noscapine was performed. The results showed that both formulations had good mean particle and aerodynamic size of 19.4 ± 6.1 nm and 3.1 ± 1.8 µm and 13.4 ± 3.2 nm and 2.3 ± 1.5 µm respectively. The cellular studies in A549 LC cells revealed that the effervescent-based formulation had enhanced cytotoxicity, apoptosis, and cellular uptake compared to the non-effervescent one. The in vivo studies were performed on Swiss albino male mice. The analysis of drug pharmacokinetics and distribution following inhalation demonstrated the superiority of effervescent-based formulation that exhibited 1.12 and 1.75-fold enhancement in drug half-life compared to non-effervescent formulation or drug powder [95].

## 6. Inhalable, Anticancer Drug-Loaded Lipid-Based Nanocarriers

**Figure 2:**
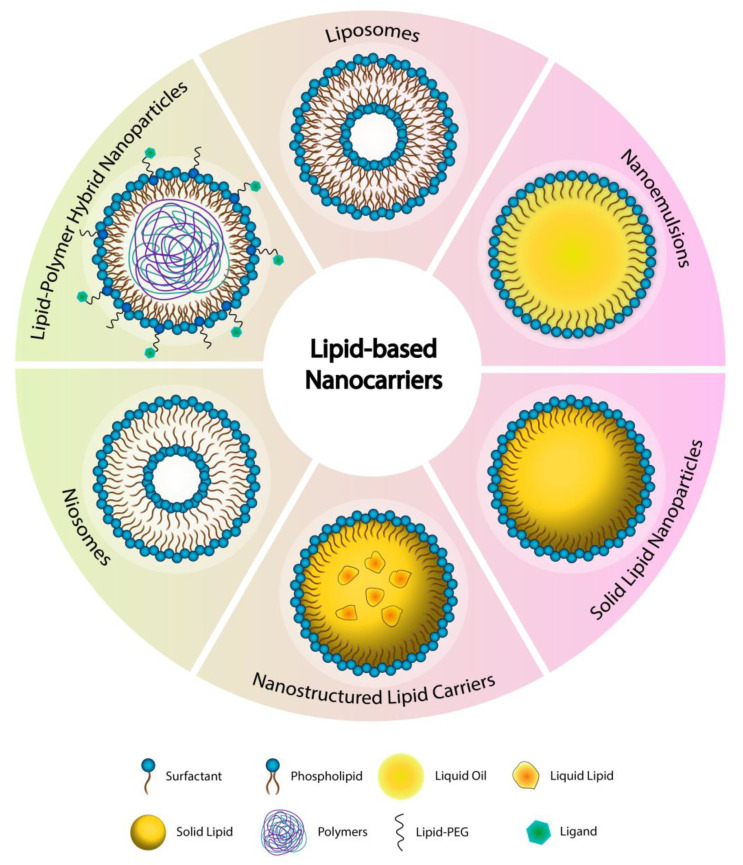
The various types of the lipid-based nanocarriers.

The lipid-based nanocarriers are gaining significant interest by researchers working on the development of novel formulations for the pulmonary delivery of anticancer drugs owing to their biocompatible, biodegradable, non-toxic, and non-irritant nature, the ability to entrap and deliver diverse molecules in a controlled manner with enhanced bioavailability, ability to transport across blood vessels and different membranes and barriers in addition to the ease of preparation and scale-up [98,99,100,101,102]. Furthermore, their surfaces are highly tunable and can be functionalized by different ligands to target the cancerous cells in the lungs. Taking into consideration that the majority of the newly discovered anticancer drugs belong to class II drugs according to the biopharmaceutical classification system (i.e., have poor water solubility and poor oral bioavailability) is turning lipid-based nanoparticles to be an excellent choice for researchers in this field. Lipid-based nanoparticles are the first type of drug delivery systems translated from principle to clinical application and now represent a well-developed, established, and evolving technology platform with significant clinical acceptability [103]. Each type of lipid-based carrier has a unique structure, as shown in Figure 2. In this review, the most recent studies about the inhalable anticancer drug-loaded liposomal formulation that include in vivo studies are discussed in the following section and summarized in Table 1. While all the published research work for the other types of lipid-based nanocarriers (i.e., nanoemulsions NEs, solid lipid nanoparticles SLNs, nanostructured lipid carriers NLCs, niosomes, and the others) are discussed in the following sections and summarized in Table 2.

### 6.1. Liposomes

Liposomes are the primary and the most widely studied systems of the lipid-based nanocarriers for the delivery of anticancer agents using different targeting strategies for the treatment of various tumors, including LC. They are first reported and described by Bangham and his colleagues in 1960 [136]. In the subsequent years, several phospholipid bilayer structures were defined, originally called bangosomes and then liposomes, as a result of combining two Greek words, “lipos” meaning fat, and “soma” signifying “body” [137]. Liposomes are self-assembled unilamellar or multilamellar spherical vesicular systems typically composed of one or more phospholipids bilayers surrounding an aqueous core (Figure 2). Liposomal properties vary considerably depending on their lipid composition, preparation method, size, surface charge and functionalization moiety. Liposomes are typically prepared using phospholipids of various origins (natural sources such as egg yolk and soybean oil, or synthetic), cholesterol and surfactants. Generally, liposomal constituents are mimicking the biological membranes and naturally present in the pulmonary surfactants that make them non-immunogenic, biodegradable and biocompatible. The size range of liposomal systems varies between 30 nm up to several micrometers [138,139]. The surface of the liposomes is highly tunable and could be functionalized using various formulation and targeting moieties. Furthermore, because of their unique structure and composition, liposomes are able to incorporate and deliver anticancer agents (such as chemotherapeutics, genes, and peptides) of highly diverse physicochemical properties and lipophilicities, where they can enhance the therapeutic efficacy by passive or active lung targeting, reduce toxicity and improve the pharmacokinetic profile of the incorporated drugs/agents [140]. All these properties turned liposomes to be excellent candidates and active area of research for pulmonary delivery and LC therapy.

Recently, inhaled hydroxycamptothecin-loaded cationic liposomes were used with concomitant intratracheally delivered sonosensitizer (5-aminolevulinic acid) for the combined chemo-sonodynamic (Chemo-STD) therapy for metastatic LC. Liposomes were prepared using the thin film method and composed of soybean phosphatidylcholine, cholesterol, and octadecylamine. The in vivo cytotoxicity studies showed that the combined Chemo-STD therapy had better cytotoxicity effects than using the hydroxycamptothecin-loaded cationic liposomes or the SDT only. The in vivo studies on metastatic LC-bearing mice showed that the highest anticancer activity was obtained using the inhaled combined Chemo-SDT than the single therapy via either inhaled or intravenously administered hydroxycamptothecin-loaded cationic liposomes or the SDT alone. The authors suggested that the synergistic effect of the inhaled chemotherapy and STD led to improved apoptosis of cancer cells and the enhanced production of reactive oxygen species [104].

Inhalable cationic liposomal formulations loaded with unmethylated oligodeoxynucleotides containing CpG motifs (CpG) and polyinosinic-polycytidylic acid (poly I:C) double-stranded RNA were also prepared recently as locally delivered immunotherapy against LC where liposomes could increase the uptake of the loaded nucleic acids by the lung phagocytes thereby the activation of toll-like receptors within endosomes. Dioleoyltrimethylammoniumpropane (DOTAP) and dipalmitoylphosphatidylcholine (DPPC) were used in the preparation of liposomes. The formulations were tested in vivo using murine B16F10 model of metastatic LC. Delayed tumor growth was observed via both agents (i.e., poly I:C and CpG). However, increased pulmonary levels of interferon-γ were observed with CpG only. Inhalation of the CpG was superior to its intraperitoneal injection to slow the growth of lung metastases and to induce the production of granzyme B, a pro-apoptotic protein, and interferon-γ, monokine induced by the gamma interferon (MIG) and the (regulated upon activation, normal T cell expressed and presumably secreted) (RANTES), T helper type 1 cytokines and chemokines, in the lungs. These antitumor activities of CpG were efficiently enhanced by CpG loading in liposomal formulations [105].

Functionalized inhalable dry powder of folic acid-conjugated liposomal formulation of docetaxel was developed for the treatment of LC [75]. The folic acid-conjugated liposomes were prepared by the thin-film hydration method and were composed of phosphatidylcholine, cholesterol, DSPE-PEG_2000_-FA, and DESP-PEG_2000_-COOH. The prepared liposomal dispersions were then co-spray dried with mannitol and leucine at different concentrations. The particle size (PS), dispersity (*Đ*), zeta potential (ZP), and entrapment efficiency (EE%) of the re-dispersed liposomes were 346.8 ± 4.7 nm, 0.401, −29.3 ± 1.8 mV, and 99.5 ± 0.3%, respectively. While the liposomal dry powder had D_ae_, FPF, spray drying production yield (PY), angle of repose (θ), Carr’s index and Hausner ratio of 3.10 ± 0.005 μm, 10.0 ± 0.1%, 61.9% ± 0.5%, 36.8 ± 0.4 °, 32.1 ± 1.86 and 1.47 ± 0.04, respectively. The morphological studies of re-dispersed liposomes showed that they were spherical as before; instead, they had irregular shapes attributed to the effects of the spray drying process. The results of in vivo studies on Sprague Dawley rats showed a 45-fold higher concentration of docetaxel in the lungs of the studied rats at 30 min after the tracheal administration compared with the intravenously administered formulation. Higher drug exposure at the tumor site was obtained by the tracheal administration of the dry powder without exposure increment to other organs. The authors concluded that the inhaled dry powders might be clinically effective for the treatment of LC [75].

Liposomal dry powder formulation of curcumin was developed as an inhalable treatment for primary LC to overcome the drug-associated drawbacks such as low water solubility, poor bioavailability, and rapid metabolism that significantly limits clinical applications. The liposomes were initially prepared using the thin film method and were composed of soybean lecithin and cholesterol. The resulted liposomes were then lyophilized in the presence of mannitol as a cryoprotectant to obtain the final liposomal curcumin dry powder. The rehydrated curcumin-loaded liposomes had PS and *Đ* of (94.65 ± 22.01 nm) and (0.26 ± 0.01), respectively. While the liposomal power had D_ae_ of 5.81 μm with FPF of 46.71%, rendering the powder suitable for pulmonary delivery. The in vitro cell culture studies showed significantly greater and faster cellular uptake of curcumin-loaded liposomes by human LC A549 cells than free curcumin. Furthermore, the high cytotoxicity of curcumin-loaded liposomes on A549 cells and their low cytotoxic activity against normal human bronchial BEAS-2B epithelial cells produced a high selection index partly due to increased cell apoptosis. The in vivo studies were performed by directly spraying curcumin liposomal powder, curcumin powder, and gemcitabine into the lungs of male Sprague–Dawley (SD)rats with LC through the trachea. Higher anticancer effects were obtained by developed liposomal curcumin powder than the other two tested medications in terms of pathology and the expression of various cancer-related markers such as VEGF, malondialdehyde, TNF-α, caspase-3, and BCL-2. Accordingly, the developed curcumin liposomal dry powder formulation has the potential to be used as inhalation therapy for LC [108].

The use of bacterial therapy is an emerging treatment technique for various cancers and may represent a promising strategy to combat LC when locally delivered by inhalation. Recently, inhaled live carriers (paclitaxel-in-liposomes-in-bacteria) were prepared and evaluated for the treatment of primary LC. The paclitaxel-load liposomes were prepared using the thin film method and composed of soy phosphatidylcholine and cholesterol. The drug-loaded liposomes were then internalized by electroporation into bacteria (*Escherichia coli* or *Lactobacillus casei*) to get LP-in-*E. Coli* (LPE) or LP-in-*L. Casei* (LPL). The PS, *Đ*, ZP and EE% of the developed paclitaxel-load liposomes were 64.3 ± 2.4 nm, 0.35 ± 0.08, −9.96 ± 0.48 mV and 97.2 ± 0.5% respectively. In vitro cytotoxicity studies on the A549 cell line revealed that LPE caused the highest inhibition of cellular proliferation compared to LPL, paclitaxel-loaded liposomes, a mixture of paclitaxel-load liposomes and bacteria. Paclitaxel-in-liposomes-in-bacteria delivered the cargos into the cells quicker than the other tested samples. The results of the in vivo studies on primary LC animal model using male Sprague–Dawley (SD) showed that among all the studied formulations, LPE had the highest anticancer effect with the downregulation of vascular endothelial growth factor (VEGF) and hypoxia-inducible factor 1-alpha (HIF-1α) and the enhancement of malignant cell apoptosis following the intratracheal administration. Furthermore, the live bacterial carriers significantly improved the expressions of (tumor necrosis factor- α, interleukin 4, and interferon-γ) immune markers and (leukocytes and neutrophils) immune cells [106].

### 6.2. Nanoemulsions

The first record in the history of nanoemulsions (NEs) began in 1943 with Hoar and Schulman [141]. However, it was not until 1993 that the term “nanoemulsions” or “ultrafine emulsion” was first reported, reflecting a formulation with a nanoscale droplet size (PS) of 20 nm to 200 nm, a transparent and semi-translucent appearance, and long-term thermodynamic stability against sedimentation by preventing flocculation, aggregation, coalescence, and Ostwald ripening [142,143]. Generally, the International Union of Pure and Applied Chemistry (IUPAC) does not yet have a fixed PS range for NEs [144], while the US FDA is considering NEs PS in the nanoscale range (approximately 1–100 nm) [145]. However, NEs prepared for pulmonary delivery must comply with the PS parameter set for this route.

NEs have gained popularity from the fact that they can be formulated from natural or synthetic excipients that are generally recognized as safe (GRAS) or approved by the US FDA [143,146,147]. Their structure is illustrated in Figure 2. In chemotherapy delivery, NEs superiority over conventional delivery systems originates from their ability to achieve the required therapeutic effect by enhancing the solubility and bioavailability of poorly soluble drugs, which may significantly contribute to decreasing drugs’ dosing and frequency as the drug is released in a sustained release manner over longer times [148,149]. Since vascularized tissues surround the cancer cells, NEs can easily accumulate in these tissues because of the small PS that gives them the advantage to pass through such barriers [150] via direct transcellular or paracellular transport. By proper selection of formulation excipients, they could have the ability to inhibit the P-gp efflux, thus enhancing cellular and mucosal permeability of the incorporated anticancer drug [118]. Besides, NEs lipophilic core is augmenting the nanosystem’s stability by protecting the drug/compound against the enzymatic hydrolysis allowing better drug delivery [151].

NEs can be categorized as simple or multiple emulsions depending on whether the core is either water or oil and the complexity of the carrier [147]. As far as pulmonary drug delivery is concerned, NEs can be classified into three generations; first-generation are prepared by spontaneous emulsification and composed of oil, surfactants, co-solvents, and a selected aqueous phase such as deionized water or saline solution [151,152]. The second-generation NEs contain the same materials as the first, but their droplets are additionally decorated with specific polymers (chitosan, hyaluronan, hydroxypropyl methylcellulose (HPMC)) to enhance mucoadhesive properties, while the third generation droplets are decorated with ligands and/or polymers for targeted drug delivery [59]. As a nonequilibrium system and a spontaneous formation is unfeasible, high energy input is applied to form NEs. This can be achieved by homogenizing the aqueous phase with an immiscible oil phase using low-and/or high-energy emulsification techniques. The size of the droplets will depend heavily on the hydrophilic-lipophilic balance (HLB) values of the NEs’ excipients [153], the type of instruments used, and their process parameters, such as time, stirring speed, temperature, and sample composition [147]. 

Although NEs may be constructed using long, medium, short-chain fatty acids or any mixture of them, however, it is noted that the inhaled NEs prepared for the delivery of conventional (non-cytotoxic) drugs were mostly composed of either medium [154,155,156] or long-chain fatty acids separately [152,157]. In contrast, inhaled NEs for anticancer delivery are usually prepared using a mixture of both (i.e., the long and medium-chain fatty acids), as illustrated in the following sections. Future studies could focus on comparing the impact of the fatty acid chain lengths on the suitability, efficiency, and biocompatibility of the inhaled anticancer NEs for lung delivery [158,159].

In addition to the oil phase, selecting a proper surfactant system is essential for the proper development of NEs for pulmonary delivery. The use of non-ionic surfactants is more prevalent than ionic surfactants in the formulation of NEs, due to the suggested deterioration of the biological membrane by their use. The superiority of non-ionic surfactants also comes from their ability to enhance poorly soluble drug dissolution, particle size, shape, and stability [160]. NEs safety is another concern that is primarily associated with the use of synthetic emulsifiers and is a key issue that needs to be addressed in particular to the adverse negative interactions between lipids and surfactants of the lung alveoli [161]. Most synthetic emulsifiers may trigger toxic symptoms with prolonged administration, including the potential binding of anionic emulsifiers to proteins, enzymes, and phospholipid membranes in the human body, resulting in various adverse reactions, such as enzyme dysfunction, protein structure modification, and membrane cell phospholipid [162]. Consequently, replacing synthetic emulsifiers and excipients with natural substitutes is one of the novelties on-demand in the construction of the NEs. Co-solvents may also be included in the formulation of NEs. Glycerol is used as the preferred co-solvent in almost every inhaled NEs for the lung delivery of anticancer drugs. This could be due to its ability to modify the aerodynamic distribution of the PS of the emitted aerosol droplets and to produce a slower dissolution rate, with the potential to modify the cell permeability of the loaded drugs, which can significantly impact their lung absorption and distribution [163,164].

Since NEs behave similarly to solutions, these formulations tend to exhibit significant improvements in their in vitro aerosolization performance when nebulized compared to other suspended nanoformulations’ types [165,166]. Although there are various solidification techniques for the production of NEs as dry powders, no dry powder of anticancer drug-loaded NEs were produced. All the developed NEs were aerosolized using nebulizers only.

NEs of docetaxel were recently formulated using biocompatible excipients for the drug pulmonary delivery to overcome the drug’s low solubility and improve its bioavailability and efficacy. A mixture of medium (lauric fatty acids and palm kernel oil esters) and long-chain fatty acid (myristic fatty acids) were used as the oil phase in these NEs. The surfactants system was composed of non-ionic (Tween 80^®^ and Span 80^®^) and amphipathic (lecithin) surfactants as they are known to be non-toxic, biocompatible, and unaffected by pH. The optimized docetaxel-loaded NEs formulation had a spherical shape with PS, ZP, and entrapment efficacy (EE%) of 94.35 ± 0.77 nm, −38.64 ± 1.43 mV, and 100%, respectively. Besides, the optimized NEs were also shown to have neutral pH, with an osmolality of (301 ± 1.00 mOsm/kg) and viscosity of (1.92 ± 0.08 cP) that are suitable for the pulmonary delivery. The optimized NEs were aerosolized using OMRON MicroAIR nebulizer and were evaluated using the Andersen cascade impactor method. The nebulized NEs showed desirable aerosolization properties for pulmonary delivery where the D_ae_ and the FPF were 3.02 ± 0.26 and 92.76 ± 0.63, respectively. The in vitro cell culture studies found that the final formulation is more selective on human lung carcinoma cells (A549) than the normal cell (MRC-5). It was concluded that the developed NEs are potential carriers for docetaxel in targeting LC via the inhalation route [118]. 

Aerosolization of NEs for pulmonary delivery for LC using docetaxel and curcumin were also reported by the same group. The NEs for both drugs (separately) were designed with a mixture of medium (palm kernel oil ester) and long (safflower seed oil) chain fatty acids and a set of non-ionic (Tween 85^®^ and Span 85^®^) and amphipathic (lecithin) surfactants, as well as glycerol as a co-solvent. Both formulations were characterized and found to have the required physicochemical and aerosolization properties suitable for inhalation [119]. 

The in-vitro aerosolization and toxicity of curcuminoids NEs for LC were investigated by Al Ayoub et al.; the formulated NEs were composed of medium (limonene) and long (oleic acid) fatty acids as oil phases, Tween 80^®^ as the surfactant, and ethanol as the co-surfactant. Based on the loaded amount of curcumin (100–500 µg/mL), the developed NEs had the PS of (13–39 nm) and *Đ* of (0.1–0.2) as well as osmolality, pH, and viscosity in the range of (336 to 600 mOsm/kg), (6–7), and (1.1–1.7 mPas) respectively. The nebulized NEs prepared with limonene oil had FPF and D_ae_ ranged from 50% and 4.6 μm to 45% and 5.6 μm, respectively; whereas the FPF and D_ae_ of the nebulized NEs prepared with oleic acid oil ranged from 46% and 4.9 μm to 44% and 5.6 μm, respectively. Genotoxicity using Comet assay showed that the developed NEs are nontoxic at the tested curcuminoid doses suggesting the safety and suitability of the developed NEs. The authors recommend further pre-clinical and clinical studies [120]. However, additional cytotoxicity evaluation and in vitro release study are also essential in such formulations.

Quercetin is a flavonoid phytochemical that is suggested to treat LC via its antiproliferative and antimetastatic effect on A549 cells through the impact on the cytoskeleton and repressing the metastatic capacity of LC via suppressing, as well as promoting apoptosis in LC [167]. NEs of quercetin were employed to enhance the lung delivery of this poorly soluble flavonoid for the treatment of LC. The in vitro cytotoxicity studies showed some selectivity of the quercetin-loaded NEs towards the A549 cells line without affecting the normal cells [121,122]. 

Although the used excipients in all these studies are considered safe, but the long-term safety studies due to possible adverse interactions with lung surfactants and efficacy of developed formulations against LC are strongly encouraged at the in vitro and preclinical level before reaching to clinical trials.

Like other lipid-based formulations, NEs that are working through passive targeting, are facing limitations in recognizing cancer or normal cells. Active targeting of the nanoemulsions could be approached by modifying the surface of these carriers, where the attached ligand (monoclonal antibodies, transferrin, folic acid, hyaluronic acid, aptamer, or antibody fragments) aids in recognition of the target tumor cells [146]. The development of anticancer-loaded NEs for active targeting decorated with ligands such as the folate-targeted NEs loaded with docetaxel [168] and transferrin-targeted docetaxel NEs [169] for ovarian cancer are already developed but currently limited for intravenous delivery. Inhaled NEs with active targeting moieties for the treatment of LC as far as we are aware, are not explored yet.

### 6.3. Solid-Lipid Nanoparticles (SLNs)

Solid lipid nanocarriers (SLNs) were introduced in 1991 as an upgrade to the traditional colloidal drug delivery systems. They are best represented as a mixture of liposomes and niosomes containing phospholipids and surfactant molecules, with a submicron PS ranging from 40 to 1000 nm [170,171]; they are derived from oil-in-water (O/W) emulsions by replacing liquid lipids with a lipid matrix that is solid at room and body temperatures [172], as illustrated in Figure 2. The use of solid lipids instead of liquid oils can result in controlled release of drugs as the mobility of the drug in a solid lipid matrix is significantly lower than that of liquid oil [173]. SLNs are composed of physiologically tolerated and safe lipids such as fatty acids (e.g., stearic acid), monoglycerides (e.g., glycerol monostearate), diglycerides (e.g., glycerol behenate), triglycerides (e.g., tripalmitin, tristearin, trilaurin), waxes (e.g., cetyl palmitate), or steroids (e.g., cholesterol) that are dispersed with an appropriate surfactant phase [174]. Next to the design of the inhalation devices, drug carrier’s selection is equally important in assuring the sufficient stability and appropriate size delivery of the loaded drug, thus, lipids and surfactants selection is an essential factor for SLNs characteristics [175]. Generally, high-pressure homogenization and microemulsion methods are being the most commonly used for the preparation of SLNs. 

Pharmacokinetically, SLNs, and liposomes have been reported to be eliminated from the lungs at comparable rates, even though SLNs are deposited after intratracheal instillation in the upper respiratory tract and, in particular, through the mucociliary escalator and do not stimulate significant inflammatory reactions [176]. The inhaled radiolabelled SLNs biodistribution showed significant uptake in lymphatics, with a high rate of distribution in periaortic, axillary, and inguinal lymph nodes and these findings indicate that SLNs may have the potential to be efficient carriers for lymphoscintigraphy or pulmonary therapy [28]. Besides, some SLNs may remain mostly intact in the pulmonary area, which may lead to longer lung retention times [177]. 

As safety and lung tolerability are of the essential parameters to be considered while developing formulations for pulmonary delivery, some studies preferred to assess the toxicity of the inhaled blank SLNs before deciding to load them with active materials. For instance, a blank SLNs formulation was designed using a lipid matrices mixture of triglycerides (Softisan^®^) and phosphatidylcholine (Phospholipon^®^ 90G), Solutol^®^HS15 as a surfactant, and double-distilled water. The high-pressure homogenization method was used for the preparation. The produced SLNs had PS, *Đ*, and ZP of 98.4 nm, 0.148, and −14.6 mV, respectively. The MTT assay and neutral red uptake assay (NRU) on the A549 cell line for 24 h showed the blank SLNs ability to reduce this cell line viability with calculated half-maximal effective concentration (EC_50_) of 3090 µg/mL and 2090 µg/mL, respectively. The organotypic cultures of lung tissue showed that the SLNs reduced the metabolic activity of the used murine precision-cut lung slices after incubating it with SLNs for 24 h and using WST-1 assay at EC_50_ of 575 µg/mL. The SLNs were nebulized by a jet-driven aerosol generator system. The in vivo cytotoxicity study on female BALB/c mice for 16 days showed no significant changes or upregulation in lactate dehydrogenase levels as an exponent of low levels of damage to the cell membrane, as well as bronchoalveolar lavage fluid protein as an indicator of cytotoxicity in lung tissues, and different inflammation indicators (TNF-a, IL-8 (A549), IL-6, and chemokine KC) [123]. Interestingly, this system was reported without loading it with an active ingredient; moreover, such evaluation should be conducted using both LC and normal cell lines to get a complete understanding of the developed SLNs’ cytotoxicity and selectivity.

Inhaled SLNs were also used to get rapid drug deposition in lungs, less systemic side effects, and improved drug therapeutic efficiency of erlotinib (a quinazoline derivative with antineoplastic properties). The SLNs were synthesized from Compritol 888 ATO^®^ (solid lipid), Tween 80^®^ (surfactant), and Poloxamer 407^®^ (an aqueous phase surfactant) using the hot homogenization method [124]. The erlotinib-loaded SLNs owned a PS (< 100 nm), *Đ* (0.367), DL% (4.17%), and EE% of (78.21%). For aerosolization of the developed NLCs, they were further spray dried in the absence and presence of mannitol (as an inert bulking agent). The dry powder of aerosolized erlotinib-loaded SLNs in the presence of mannitol had D_ae_ (3.93), emitted dose (ED)% (94.91), FPF% (30.98), and geometric standard deviation (GSD) of (4.339). The TEM and scanning electron microscopy (SEM) micrographs of both liquid and powder SLNs indicated a regular and spheroidal shape with smooth surfaces. The in vitro release studies using the dialysis membrane method showed that here was no burst release from the formulated SLNs and cumulative drug release occurred with a steady rate to reach approximately 12% at 8 h, as compared to ~18% with free drug. Besides, the MTT assay revealed significantly higher anticancer activity of the erlotinib-loaded SLNs against A549 cells in comparison to the free drug and after 18 h of incubation. However, no in vivo studies were performed for the elevation of organs distribution and anticancer activity were performed in this study.

Epirubicin which is an anthracycline and a stereoisomer of doxorubicin that has shown activity against various types of tumors including LC, but its use is associated with major side effects including hematological and cardiac toxicity, thus specific targeting through simple, safe and stable formulations is highly recommended. Accordingly, Epirubicin-loaded SLNs were prepared. The SLNs were composed of soy lecithin, compritol 888 ATO^®^, and poloxamer 188^®^. The produced SLNs had the characterization of PS, ZP and EE% of 223.7 nm, −30.6mV, 78.9% respectively. The formulation was nebulized using (Pari Inhalierboy, Starnberg, Germany). No significant changes in PS ZP or EE% were observed after nebulization [125]. The nebulized formulations were evaluated for their in vitro deposition by a Twin Stage Impinges (TSI). The blank SLNs, epirubicin-loaded SLNs and pure epirubicin solution showed respirable fractions (RF) of 77.03%, 78.46%, and 59.51%, respectively indicating the decrease in drug loss, besides the SLN possible ability to deliver the drug into the deep lung. The cytotoxicity on A549 cells using 0.1% crystal violet after incubation for 24 h revealed the improved cytotoxic effects of the developed SLNs in comparison to the free drug. Pharmacokinetically, and upon analyzing plasma and lung samples via HPLC, aerosolized epirubicin-loaded SLNs showed excellent lung deposition characteristics compared to epirubicin solution in male Sprague–Dawley rats, while the plasma area under the curve values for epirubicin-loaded SLNs was 2.07-fold higher than that after epirubicin solution suggesting the potential suitability of the developed inhaled SLNs for pulmonary delivery to treat LC.

SLNs were employed also for the co-delivery of afatinib and paclitaxel for the treatment of epidermal growth factor receptor tyrosine kinase resistant NSCLC. In this study, afatinib was first loaded in SLNs composed of stearic acid and poloxamer 188 and had PS, *Đ*, and EE% of 358.3 nm, 0.167, and 87.9% respectively. Furthermore, these SLNs were lyophilized using trehalose (as a cryoprotectant) and loaded with paclitaxel into poly-lactide-co-glycolide-based porous microspheres. These inhaled microspheres systems are characterized with D_ae_, FPF, fine particle dose (FPD), and GSD of 3.26 and 3.25 µm, 23.04 and 24.07%, 41.01 and 59.66 µg, 2.26 and 2.32, as well as EE% 53–70.85% of afatinib and paclitaxel, respectively. These final formulations showed an initial in vitro drug release for paclitaxel (20%) and afatinib (30%), with extremely high retention (more than 65%) in the induction port (17.21 ± 0.22% for afatinib and 16.00 ± 1.52% for paclitaxel), and no interaction between drugs and carriers when characterized by FTIR and NMR spectroscopy [126]. On the cellular level, there was a significant synergistic effect between afatinib and paclitaxel and superior treatment capability of the final loaded microspheres for drug-resistant NSCLC on H1975 and PC9/G cells. The pharmacokinetics and tissue distribution results demonstrated that afatinib and paclitaxel in the microspheres exhibited 96 h of a two-stage release and high lung concentration. The final loaded microspheres did not distribute to other critical organs. These results revealed that the drug combination therapy using these nanocarriers is highly promising for treating drug-resistant LC.

### 6.4. Nanostructured Lipid Carriers (NLCs)

The nanostructured lipid carriers (NLCs) represent an advanced type of the SLNs. These carriers can overcome the SLNs-related disadvantages, such as the drug loading capacity and formulation stability challenges by creating a less structured solid lipid matrix via mixing fluid lipid with solid lipid (as shown in Figure 2), resulting in less drug expulsion during storage [173,178,179]. NLCs are the products of o/w emulsion process, hence the available surfactants typically have a high HLB range, and ideally dissolved in the external aqueous phase of the emulsion [160]. Besides, these nanocarriers can be used to circumvent the limitations associated with conventional cancer chemotherapy such as poor drug solubility, and multiple drug-resistance by enhancing chemotherapy’s targeting and selectivity index [180,181]. Additionally, NLCs are suitable to carry drugs with different physicochemical properties, natural compounds and small interfering RNA (e.g., siRNA), where the latter is currently trending as an NLCs conjugate due to its proved ability in recognizing a homologous mRNA sequence in the cancer cell and induce its degradation [182]. In this regard, and chemistry wise, a smooth conjugation between thiol-modified DNA or RNA molecules (e.g., siRNA) and the NLCs surface occurs by biodegradable disulfide (S–S) bonds. Further conjugations with NLC include polymers conjugation (e.g., PEG) with targeting fractions (e.g., luteinizing-hormone releasing hormone (LHRH) peptide) [183,184]. However, the key drawback of NLCs is the need to use organic solvents to initially solubilize the hydrophobic drugs before loading [185], as well as the short-term stability of the liquid NLCs compared to the solid ones [186,187]. Like the previously discussed lipid-based nanocarriers, the use of NLCs as localized inhaled dosage forms is still under investigation mainly as active carriers for anti-tuberculosis [188], genetic disorders such as lung cystic fibrosis [189], antibiotics lung delivery [190,191], in addition to LC therapies. In LC, NLCs are often used to resolve p-glycoprotein (P-gp) efflux, and drug resistance which is generally associated with over-expression of MRP1 protein (responsible for cancer cell drug efflux) and BCL2 protein (responsible for anti-apoptotic cellular defense) [192,193,194,195].

Inhaled NLCs were used for the pulmonary delivery of various drugs and approaches for LC treatment. The cyclooxygenase-2 enzyme, which is responsible for the progression and growth of NSCLC and found to be up-regulated among different cancers [196,197]. Thus, the efficacy of inhaled celecoxib-loaded NLCs in NSCLC in combination with IV administered docetaxel was evaluated using a metastatic A549 tumor model in Nu/Nu mice. The NLCs were initially prepared using a hot melt homogenization technique via mixing compritol (solid lipid), miglyol (liquid lipid), and sodium taurocholate (surfactant). The PS, *Đ*, ZP, drug content, DL, EE% of the NLC produced were 211 nm, 0.22, 25.30 mV, 1.8 mg/mL, 4 *w/w*%, and 95.6%, respectively. The celecoxib-loaded NLCs were nebulized using Inexpose™ (SCIREQ Scientific Respiratory Equipment Inc, Montreal, QC, Canada). The aerosolized NLCs had D_ae_ and FPF were 1.58 μm and 76.2%, respectively. The isobologram of the interaction between docetaxel and celecoxib-NLC in the A549 NSCLC cell line suggests moderate synergistic activity. While the analysis of the 28-days in vivo studies showed that treatment with inhaled celecoxib-NLC, IV docetaxel, and the combination of both treatments decreased tumor volume by 25%, 37%, and 67%, respectively, without a substantial decrease in mice weight compared to control group. Besides, the inhaled celecoxib-NLCs, IV docetaxel, and combined therapy have also decreased vascular endothelial growth factor expressions in regressive tumors by 0.27, 0.44, and 0.65 times, respectively, compared to control. The quantitative proteomic analysis shows a significant reduction in the regulation of multiple proteins demonstrating enhanced anticancer activity in combination therapy compared to docetaxel treatment alone [128].

A comparison in lung deposition was evaluated in vivo using Wistar rats between pulmonary delivered paclitaxel loaded-NLCs (as a dry powder delivered using insufflators (Penny Century, PA, USA)) and orally administered methanolic PBS suspension of the drug [129]. The NLCs were prepared by the emulsification and ultrasonication method using various surfactants. The solid and liquid lipids phase consisted of stearic acid (or glyceryl monostearate) and oleic acid at different concentrations, while the aqueous phase was composed of different amounts of Tween 80^®^, Tween 20^®^, or Tween 40^®^. The statistical analysis showed that the low lipid ratio, the high levels of surfactant concentration and, the medium homogenization speed provided favorable ranges of PS, *Đ*, and ZP values for Tween 20^®^ (178.7 nm, 0.158, −15.22 mV), Tween 80^®^ (243.1 nm, 0.225, −16.12 mV), and Tween 60^®^ (298.2 nm, 0.281, −22.23 mV). The NLCs formulated with Tween 20^®^ showed the highest uptake of Caco-2 cells, which could be attributed to Tween 20^®^ ability to inhibit P-gp efflux [198]. As a result, the Tween 20^®^-based NLCs were further spray-dried using leucine as anti-adherent to produce NLCs powder with PS, *Đ*, ZP, and an in vitro release of 283.4 nm, 0.226, −25.12 mV, 64.9%, respectively. The dried NLCs had good powder and flow properties with a D_ae_ of 3.53 μm. Lungs’ uptake of the drug from the powdered NLCs was higher than the plain drug suspension. This could be attributed to the less clearance of the drug from the lungs due to the slow release of the drug from the NLCs and the retention of the drug in lipid nanoparticles. This indicates the superiority of local delivery via the pulmonary route [129].

The concept of multifunctional NLCs-based delivery systems substantially enhanced the efficiency of NSCLC therapy with suggested abilities to limit the adverse side effects of the treatments, primarily when targeting strategies are used and administered via inhalation. In this regard, multifunctional anticancer (doxorubicin or paclitaxel) and siRNA-loaded NLCs for pulmonary delivery via nebulization were developed for the treatment of LC. The NLCs were functionalized with a modified synthetic analog of luteinizing hormone-releasing hormone (LHRH) as a targeting moiety. In addition, they were conjugated with (1,2-Distearoyl-sn-glycero-3-phosphoethanolamine-poly(ethylene glycol) (DSPE- PEG). The developed doxorubicin-NLCs were primarily used to evaluate cellular uptake and the intracellular localization due to the intrinsic fluorescence of doxorubicin, while the paclitaxel-NLCs were used to assess the anticancer efficacy of the formulation. After the preparation process, the final NLC was purified via dialysis (MWC 10,000) and lyophilized with mannitol (5%) as a cryoprotectant. In vivo orthotopic model of human LC in nu/nu mice was used to evaluate the anticancer activity and tissue distribution. After inhalation, the developed NLCs efficiently delivered their payload into LC cells, leaving healthy lung tissues unaffected compared with IV injection. The tumor size decreased from 117 mm^3^ to 20.8 mm^3^ and 2.6 mm^3^ upon treatment with LHRH-NLC- paclitaxel, and LHRH-NLC- paclitaxel -siRNAs, respectively. The obtained results showed the high efficiency of the inhaled NLCs for tumor-targeted local delivery, specifically LC cells. As a result, effective suppression of tumor growth and prevention of adverse side effects on healthy organs [130]. 

The same concept in the latter study was used recently to developed paclitaxel tumor-targeted NLCs using the melted ultrasonic method after successfully mixing Precirol ATO 5^®^ (solid lipid), squalene (liquid lipid), and soybean phosphatidylcholine (emulsifier) with the aqueous phase, which was composed of Tween 80^®^ (surfactant) and (N-[1-(2,3-dioleoyloxy)propyl]-N,N,N-trimethylammonium) “DOTAP” (a cationic lipid which grants positive charge to NLC) in deionized distilled water, while paclitaxel was dissolved in dimethyl sulfoxide (DMSO). PEG2000 was the most suitable choice for the linkage of LHRH peptide with the NLCs, and later it was further conjugated with siRNA. The LHRH-NLC-siRNAs-paclitaxel-loaded nanoparticles had distinct spherical shape with PS, ZP, and loading efficiency of 113 nm, +45 mV, and 98%, respectively. On the cellular level, the toxicity of the developed formulation was superior to the traditionally available epidermal growth factor inhibitor, gefitinib, in three types of cells, including H1781, H3255, and A549 cells lines, as such sensitivity was linked to the presence of LHRH. The in vivo study was performed using an orthotopic NSCLC mouse model. The NLCs formulations were administered via IV and inhalation (using a Collison nebulizer (BGI, Inc., Waltham, MA, USA) methods. The results showed that developed multifunctional NLCs had a suggested efficient accumulation and retention in the lungs when inhaled compared to the IV route. The immunoperoxidase assay indicated that the formulation did not induce an immune response in human peripheral blood lymphocytes. Besides, no signs of toxicity were observed (in vivo) in the (liver, kidney, spleen, heart, lung, brain) of nude mice following inhalation or IV administration [131].

In summary, NLCs are potential carriers for the pulmonary delivery of anticancer drugs, they were successfully developed for this purpose using GRAS materials. They have the advantage to be efficiently functionalized using different ligands for active targeting. Besides, they can be aerosolized using nebulization or converted to dry powders to be used in DPIs. The results from the in vitro and in vivo studies are highly promising in the treatment of LC. However, these lipid-based nanocarriers were not tested in any clinical trial yet.

### 6.5. Miscellaneous Inhaled Lipid-Based Nanocarriers

A number of certain types of lipid-based nanocarriers were addressed for the delivery of anticancer for the treatment of LC via inhalation is available but at a very limited scale. Among these are the lipid-polymer hybrid nanoparticles (LPHNs), these nanocarriers incorporate the advantages of both liposomes and polymeric nanoparticles into one novel drug delivery platform [199]. LPHNs are typically consist of a biodegradable polymeric hydrophobic core and an outer shell made of lipid or lipid-ligand [200] (Figure 2). LPHNs may offer some benefits, such as physical stability and biocompatibility; their surfaces are highly tunable so they are suitable for the passive and active drug targeting, they also provide controlled release of drugs [201]; reduced systemic toxicity; and therefor they can potentially enhance efficacy of anticancer drugs [202]. However, despite all these potential advantages, these nanocarriers are not well explored for the pulmonary delivery. 

In one study, LPHNs were used in the downregulation of genes involved in the pathogenesis of severe lung diseases such as LC through the local siRNA delivery. The developed LPHNs were composed of poly(lactic-co-glycolic) acid and dipalmitoylphosphatidylcholine as siRNA inhalation formulation and prepared using the emulsion/solvent diffusion method. The optimized formulation was found to have PS and ZP in the range of (135 to 169 nm) and (−16 to −30 mV), respectively, with *Đ* < 0.130 and EE% of 75%. The formulation possessed a peculiar triphasic release profile, characterized by an initial burst, with more than 50% of siRNA released in the first hours, followed by a slow-release phase lasting a couple of days and a final fast release time period after 4–5 days. The nebulized formulation was having D_ae_ < 5.39 µm. Before each experiment, freeze-dried HLPNs were dispersed in 0.5 mM sodium chloride. The stability of the developed siRNA-loaded LPHNs was confirmed by TEM analysis of freeze-dried formulation in the presence of mannitol before and after nebulization in the Vitrocell Cloud system (Vitrocell Systems GmbH, Waldkirch, Germany). On the cellular level, these LPHNs were able to penetrate into cells effectively and are localized intracellularly on the TCCC cell line leading to an effective in vitro gene silencing (on A549 cells line) in the form of knocking down both aENaC and bENaC subunit proteins up to 72 h. The developed nanosystem was muco-inert and stable inside artificial mucus with no cytotoxic or acute proinflammatory effect toward any of the cell components of the co-culture model. The results demonstrated the high potential of using HLPNs as carriers for pulmonary delivery of siRNA [132]. 

These hybrid nanocarriers are having excellent potential for the pulmonary delivery of drugs, and their possible role in delivering inhaled anticancer drugs is not well investigated and could be considered for future studies in this field.

Niosomes are also among the systems that are rarely investigated for inhaled anticancer therapy to treat LC. Niosomes are also known as non-ionic surfactant-based vesicles (Figure 2). These carriers gained much interest in the pharmaceutical field due to their excellent abilities to encapsulate and efficiently deliver drugs/agents of different physicochemical properties via different routes of drug administration. Furthermore, their production is easy to scale up at low costs. Besides, these nanoparticles demonstrated to be more stable than liposomes during the formulation phase or upon storage. The required pharmacokinetic properties can be achieved by optimizing the components or modifying the surface of niosomes [203]. Particle size and zeta potential are essential to the pharmacokinetics, bio-distribution, toxicity, and stability of niosomes and should be well considered [204,205].

Inhalable cationic niosomes of curcumin were developed for effective and local delivery to LC cells [134], to circumvent the poor physicochemical and biopharmaceutical limitations of curcumin associated with its oral and parenteral administration, such as the poor and unpredictable bioavailability at the site of action and the extensive first-pass metabolism and irregular bio-distribution [206,207]. The developed niosomes were prepared using the reverse-phase evaporation method and composed of span 80^®^, diethyl ether, and chloroform with or without cholesterol. The prepared formulations were further freeze-dried using mannitol as a cryoprotectant. The resulted curcumin-loaded niosomes (containing cholesterol) (Cur-C-SUNS) were cationic and unilamellar with PS (97.4 nm), ZP (+28.5 mV), and %EE of (83.3%). While the freeze-dried niosomes prepared without cholesterol (Cur-SUNS) had a smaller PS (83.8 nm), and ZP value of (−3.02 mV), and EE% of (78.8%). The in vitro release of the powdered niosomes using dialysis membrane technique was enhanced by (30.1%), which could be due to the amorphization of nanovesicles that ultimately enhanced the solubility and release rate of the drug [208]. The optimized formulation (Cur-C-SUNS) was able to inhibit the A549 cells proliferation at the IC50 of 3.1 μM, which is significantly lower than 7.5 μM for Cur-SUNS and curcumin dispersion (< 32 μM). The in vitro cellular uptake results illustrated higher endocytosis of Cur-C-SUNS as compared to Cur-SUNS due to electrostatic interaction between cationic nanovesicles and negatively charged plasma membrane of A549 cells [134]. Although the obtained in vitro results were promising, no further in vivo studies were performed to investigate the potential roles of inhaled niosomes for the delivery of anticancer drugs for LC treatment.

Sterosomes, are new and promising non-phospholipid type of liposomes drug delivery nanoparticles, typically they composed of stearylamine and cholesterol. They are named as ‘sterosomes’ owing to their high sterol content. These carriers are highly tunable and suggested to have better stability and longer circulation and residence time than the classical liposomes [209,210].

Sterosomes were recently reported to deliver the widely used antidiabetic drug (metformin) as an inhalation dosage form because it was shown to have anticancer activities via inhibition of cellular proliferation of many cancers, including LC. The safety, tolerability and pharmacokinetics of inhaled metformin sterosomal formulation or solution. In this study, cholesterol was mixed with stearylamine or myristic acid followed by dissolving accurately weighed quantities of the solid chemicals in a mixture of benzene/methanol. The PS, %EE and ZP of the developed formulation have ranged approximately from 288.7 to 578 nm, 71% to 89% and + 16.2 to + 63.2 mV, respectively. The *Đ* values were generally <0.4. The measured D_ae_, GSD and FPF values for aerosolized metformin-loaded sterosomes by jet nebulizer were 3.3 µm, 2.114, 62.36 respectively. The MTT assay on A549 cell lines (for 48 h) showed that survival rate after exposure to metformin-containing sterosomes was very low <50%. The clinical study in this work (3 females: 3 males) at average age of 32 years old showed that the volunteers noted the greasy and ammonia-like smell of metformin sterosomal preparation. The entire process of aerosol administration of the prepared metformin-loaded sterosomes and metformin solution was generally feasible and well tolerated. The metformin-loaded sterosomes enhanced the half-life, area under the curve, and mean residence time of metformin in all healthy volunteers after inhalation of a single dose of 750 mg of metformin sterosomal formulation [135]. Authors have addressed some limitations of this study such as the relatively small number of subjects which could lead to improper variability in the results, and the lack of comparison between multiple and different doses. More extensive clinical trials with long-term follow-up are needed to confirm the safety and efficacy of the developed formulation.

## 7. Inhalable Anticancer Drug-Loaded Lipid-Based Nanocarriers in Clinical Trials

Despite the proven advantages offered by inhalable anticancer therapy via lipid-based nanocarriers in preclinical studies, the number of conducted clinical trials is still limited (Table 3). In addition, the most advanced development of inhaled anticancer therapy was performed up to phase II only; consequently, no inhaled lipid-based nanocarrier product reached the market yet. This could be attributed to the associated challenges of using this route of administration, as discussed in Section 3 of this review.

Among the various types of inhalable lipid-based nanocarriers, only liposomes were evaluated in clinical trials. The safety and pharmacokinetics of aerosolized sustained-release lipid inhalation targeting (SLIT) of cisplatin in patients with lung carcinoma were investigated. Seventeen patients and one tracheostomy patient on compassionate use received treatment. The results showed that the aerosolized liposomal cisplatin was well tolerated. In addition, no DLT was observed at the maximum delivered dose. Safety data showed that no hematologic toxicity, nephrotoxicity, ototoxicity, or neurotoxicity was observed. Pharmacokinetically, very low plasma platinum levels were obtained only with the longest repeated inhalations. The aerosolized cisplatin-loaded liposomal formulation was found to be feasible and safe [30]. 

To evaluate the safety and efficacy of inhaled lipid cisplatin (ILC) in patients with recurrent osteosarcoma who only had pulmonary metastases, an open-label, phase Ib/IIa study was performed (NCT00102531). The study involved nineteen patients. The results showed that no patients experienced hematologic toxicity, nephrotoxicity, or ototoxicity. The inhaled liposomal cisplatin was well tolerated in heavily treated osteosarcoma patients. In addition, the typical toxicities associated with intravenous cisplatin did not appear with the inhaled therapy [31,32]. A phase II clinical trial to establish whether treatment with inhaled liposomal cisplatin (ILC) formulation is effective in delaying/preventing pulmonary relapse in osteosarcoma patients in complete surgical remission following one or two prior pulmonary relapses was completed in 2018 (NCT01650090). However, no data have been published yet [211].

Aerosolized 9-nitro-20(*S*)-camptothecin (9NC)-loaded liposomal formulation was evaluated clinically for safety and feasibility in a group of 25 patients with primary or metastatic LC. The patients received the aerosolized liposomal formulation for five consecutive days/week for 1, 2, 4, or 6 weeks followed by two weeks of rest to determine feasibility. As mentioned previously, chemical pharyngitis was the DLT at 26 mg/kg/day. After inhalation, 9NC was absorbed in a rapid and sustained manner through the lung parenchyma to the bloodstream circulation. Stabilization occurred in 3 patients with primary LC, and partial remissions were observed in 2 patients with uterine cancer. The results revealed that the pulmonary administration of 13.3 mg/kg/day of the 9NC-loaded liposomal formulation was feasible and safe. Furthermore, the researchers recommended a dose of 13.3 mg/kg/day of the liposomal formulation for phase II of the study [29].

There is other six clinical trials of aerosolized 9NC-loaded liposomes have been completed but no results were released or published to the author’s best knowledge, and they are namely: (NCT00492141) for determining the effectiveness of L9NC given by aerosol in combination with temozolomide in patients with solid tumors involving the lungs (Phase II, completed in September 2009) [212], (NCT00249990) for determining efficacy and toxicity profile in metastatic or recurrent endometrial cancer (Phase II, completed in September 2007) [213], (NCT00250016) to determine the amount of aerosolized drug in patients’ blood and tumor (completed in August 2007) [214], (NCT00250068) to determine the overall response rate to 9NC administered by aerosolization in patients with NSCLC any stage (Phase II, completed in December 2007) [215], (NCT00277082) to determine the concentration of the drug in the alveolar fluid over time (completed in June 2005) [216], (NCT00250120) to determine the overall response rate to the inhaled liposomal drug in patients with NSCLC at any stage (withdrawn, Phase II, completed in August 2007) [217].

With the new advancements in the fields of lipid-based nanocarriers, drug targeting, and pulmonary delivery devices, clinical studies are currently needed to reveal the promising potentials of inhalation chemotherapy.

## 8. Conclusions

Inhalable anticancer therapy via lipid-based nanocarriers is an exciting and growing research area. It is a promising treatment strategy to combat LC and lung metastases. Due to the unique properties of the lipid-based nanocarriers of great biocompatibility, high drug loading, and tunable surfaces for active targeting and controlled drug-release behavior, they are gaining much interest. Results from the recent studies on preclinical levels revealed that the drugs loaded in these inhalable nanocarriers will be concentrated in the lungs and then diffuse gradually into the blood circulation and the lymphatic system to target the cancerous cells. Among the currently available pulmonary devices, only nebulizers and DPIs are potentially suitable for the efficient delivery of these nanoparticles. The use of DPIs as devices for inhaled anticancer drugs loaded in lipid-based nanoparticles is quite promising as they have many advantages and could overcome the challenges associated with this route. Combining the use of lipid-based nanocarriers, DPIs devices, particle engineering, and formulation sciences opens the door for new advancements and possibilities. However, the research in this field is still in its infancy, particularly at the in vivo and clinical studies levels. The general formulation strategy should concentrate on developing uni- or multifunctional lipid-based nanocarriers for active targeting, with good drug loading and sustained release properties, embedded in well-engineered microparticles composed of safe and well-tolerated excipients of high FPF for efficient lung deposition, drug delivery, and antitumor activity.

## Figures and Tables

**Table 1:** Summary of the recently published in vivo studies about the inhalable anticancer drugs-loaded liposomal formulations for the treatment of LC.

Drug/Agent	Composition	Aim	Targeting Moiety/ Strategy	Form	Delivery Method/ Device	Cell line/Species	Main Outcomes	Ref.
HC & 5-Aminolevulinic acid	SPC, cholesterol, & octadecylamine	Chemo-sonodynamic therapy for metastatic LC	Cationic liposomes	Liquid	Intratracheal/Insufflator (IA-EC, Penn-Century, Inc., USA)	Formulation evaluation on metastatic LC-bearing mice: Female Balb/c mice (19–21g).	Synergistic effect of the inhaled chemotherapy and sonodynamic therapy led to improved apoptosis of cancer cells	[104]
CpG & Poly I:C	DOTAP & DPPC	To locally deliver immunotherapy against LC	N/A	Liquid	Intratracheal instillation	Tumor growth evaluation using murine B16F10 model of metastatic LC, Specific-pathogen-free female C57BL/6 Nrj mice (age, 6–8 weeks).	Delayed tumor growth caused via both agents. Inhalation of the CpG was superior to its intraperitoneal injection in slowing the growth of lung metastases with enhanced antitumor activities.	[105]
Paclitaxel	Soybean lecithin & cholesterol.	The investigation of delivering locally live carriers (paclitaxel-in-liposomes-in-bacteria) to combat LC.	Dry liposomes internalized into bacteria (*E. coli* or *L. casei*)	Liquid	Intratracheal instillation	Anti-cancer effects evaluation using male SD rats (180–220 g)	Liposomes in E-coli: highest anticancer effect, with the downregulation of VEGF and HIF-1α and the improvement of cancer cell apoptosis	[106]
Curcumin	Lecithin, cholesterol, stearylamine, poloxamer 188, 2-hydroxypropyl-β-cyclodextrin	To overcome the curcumin poor aqueous solubility and oral bioavailability	N/A	DP	Intratracheal instillation	MTT assay on A549 Cell line. Pharmacokinetic studies using Albino rats (220–260 g)	The liposomes formulation surpassed curcumin powder in the rate and extent of lung tissue absorption and mean residence time within the lung tissues.	[107]
Curcumin	Soybean lecithin & cholesterol	To evaluate the efficacy of curcumin-loaded liposomes	N/A	DP	Insufflator (DP4M, Penn- Century Inc., USA).	MTT assay On BEAS-2B, A549 cells line. Anti-cancer activity evaluation using male SD rats (190–200g).	Liposomes curcumin dry powder showed higher anticancer effects and selectivity than free form.	[108]
Lenvatinib-bound to magnetic iron oxide NP	N/A	To investigate the use of inhaled liposomes encapsulating targeted NP contrast agent (TNCA) for diagnosing purposes	Lenvatinib	Liquid	Atomizer	Tomography studies using C57BL/6 mice both sexes (age, 6–8 weeks).	The sensitivity and accuracy of computerized tomography imaging for the diagnosis of early-stage NSCLC was improved.	[109]
Doxorubicin	DPPC, Poloxamer 188	To formulate thermosensitive doxorubicin-loaded liposomes	Hybrid liposomes	Liquid	Intratracheal administration	WST assay on A549 and Raw 264.7 cells lines. The evaluation of lactate dehydrogenase activity and tumor necrosis factor alpha secretion in cell-free bronchoalveolar lavage fluid using Wistar rats (male, age, 13 weeks).	The formulated liposomes administered via the pulmonary route maybe useful for treating LC.	[110]
Doxorubicin & ASO, or siRNA	DOTAP & cholesterol.	To evaluate the use of a nose-only exposure chamber for inhalation and delivery of doxorubicin or nucleic acids	ASO, or siRNA	Liquid	One-jet Collison nebulizer (BGI Inc., Waltham, MA, USA)	Evolution of formulation’s distribution and tumor growth size reduction using Nude nu/nu mice (age, 6–8 weeks).	The developed formulation inhalation resulted in tumor volume reduction of more than 90%, whereas only about 40% reduction was achieved after intravenous injection of the free drug.	[111]
Doxorubicin	DSPC, DSPE-PEG2000, DSPE-PEG-COOH, & cholesterol	To investigate the efficacy of active drug targeting via TF receptor-mediated uptake.	TF- PEG liposomes	Liquid	An AeroProbe intracorporeal nebulizing catheter connected to a catheter control unit	Tumor induction evaluation using female athymic Rowett nude (rnu) rats (age, 8–10 weeks).	More animals survived in the TF– liposomes groups than in the other treatment regimes, and their lung tissue generally had fewer and smaller tumors.	[112]
Quercetin	SPC, DSPE-PEG2000, cholesterol & DSPE-PEG2000- MAL-T7 conjugate	To augment therapeutic efficacy of quercetin-targeting TF receptors.	T7 (HAIYPRH) peptide	Liquid	Microsprayer^®^ Aerosolizer Pulmonary Aerosol- Kit for Mouse (Penn-Century Inc., PA, USA)	MTT assay on A549 and MRC-5 cells lines. Apoptosis, cell-cycle analysis, cellular uptake, and tumor-spheroid penetration and inhibition studies on A549 cell line. Biodistribution study and therapeutic efficacy using male BALB/c nude mice (age, 7–8 weeks).	The developed formulation significantly enhanced the anticancer activity of the drug and lifespan of mice.	[113]
Triptolide	SPC, DSPE-PEG2000, DSPE-PEG2000-MALCPP33	To explore the pulmonary delivery of dual-ligand modified and triptolide-loaded liposomes modified triptolide-loaded liposomes	Anti-CA IX antibody & CPP33 dual ligands	Liquid	Microsprayer Aerosolizer Pulmonary Aerosol Kit for Mouse Model PAK-MSA	Wound healing, apoptosis, penetration, and cytotoxic damage in 3 D tumor spheroids on A549 cell line. Pharmacokinetic study using male SD rats (250 ± 20 g)	The formulation significantly enhanced the anticancer efficacy of the drug without apparent systemic toxicity.	[114]
Docetaxel	PC, cholesterol, DSPE-PEG-FA/DSEP-PEG-COOH/Co-spray	To compare the physicochemical properties, and antitumor activities of different targeted liposomal formulations	Folic acid conjugate	Liquid	Intratracheal administration	MTT assay, cellular uptake, endocytic routes study and metabolism assay on A549 and SPCA1 cells lines. bio-distribution studies using SD rats (180–220 g).	The co-spray drying did change the properties, while tracheal administration of the dry powder provided higher drug exposure at the tumor site without increasing the exposure of other organs	[75]
Temozolomide	PC, cholesterol & auric tetrachloride	To investigate the possible therapeutic effects of intratracheal inhalation of the developed liposomes-gold NP	N/A	Liquid	Intratracheal administration using Microsprayer IA-1C system (Penn-Century, Philadelphia, PA, USA)	Study the developed formulation’s effects on lung homogenate MDA, GSH and inflammatory cytokines as well as on serum CYFRA 21-1 and IGF-1 level using male BALB/c mice (22–30 g).	The developed liposomes formulations succeed to improve all biochemical data and histological patterns.	[115]
Gemcitabine-HCl	HSPC, DSPG, mPEG2000-DSPE.	To formulate and evaluate gemcitabine-HCl -loaded liposomes	PEG- liposomes	DP	Intratracheal administration	MTT assay and cellular uptake on A549 cell line. Maximum tolerated dose, oedema index, acute toxicity study, and pharmacokinetic studies using Wistar rats (200–220 g).	Better pulmonary pharmacokinetic profile of the loaded formulation with lower toxicity to lung tissues than that of drug solution	[116]
Vincristine	SPC, & cholesterol.	To improve efficacy, lung exposure and decrease the clearance of the drug	N/A	DP	Intratracheal administration	MTT assay on MCF-7 and A549 cells lines. Absorption and tissue distribution study male SD rats (250 g).	The developed formulation had improved pharmacokinetic behavior of increased maximum concentration and systemic exposure and decreased elimination half-life in comparison to the free drug.	[41]
Gefitinib	SPC & cholesterol.	Comparative study of intratracheally administered of gefitinib- liposomes via intratracheally and orally administered free drug	N/A	DP	Intratracheal administration	Pharmacokinetic and biodistribution study using male SD rats (180–200 g).	Intratracheally administered liposomal powder showed higher in vivo therapeutic effect with reduction of inflammation, weak lung injury, and high apoptosis than intratracheally or administered free drug.	[117]
Sorafenib tosylate	Phospholipon 90H^®^ & cholesterol	To enhance the physicochemical properties of sorafenib tosylate	N/A	DP	Revolizer device (Cipla Inc.)	NA	The loaded formulation offered biphasic release pattern, burst release in the first 6 h followed by sustained release up to 72 h	[73]

Anti-CA IX: Anti-carbonic anhydrase IX; ASO: Antisense oligonucleotides; CpG: Unmethylated oligodeoxynucleotides containing CpG motifs; DOTAP: Dioleoyltrimethylammoniumpropane; DP: Dry powder; DPPC: Dipalmitoylphosphatidylcholine; DSPC: Distearoyl-sn-glycero-3-phosphocholine; DSPE-PEG2000: Glycero-3-phospho-ethanolamine-N-[methoxy(polyethylene glycol)-2000]; DSPE-PEG-COOH: Glycero-3-phosphoethanolamine-N-[carboxy(polyethylene glycol)-2000]; FA: Fatty acids; HC: Hydroxycamptothecin; HSPC: Hydrogenated soy phosphatidylcholine; NP: Nanoparticles; PC: Phosphatidylcholine; Poly I:C: polyinosinic-polycytidylic acid double-stranded RNA; SD: Sprague–Dawley; siRNA: Small interfering RNA; SPC: Soy-phosphatidylcholine; TF: Transferrin.

**Table 2:** Summary of all the published preclinical studies on the developed anticancer drugs-loaded lipid-based nanocarriers for the treatment of LC.

Drug/Agent	Composition	Aim	Targeting Moiety/ Strategy	Form	Delivery Method/ Device	Cell line/Species/Subjects	Main Outcomes	Ref.
**NEs**								
Docetaxel	PKOE, lauric FA, myristic FA, lecithin, Tween 85^®^, Span 85^®^, & glycerol	To select biocompatible excipients and perform aerodynamic characterization of nebulized NEs.	N/A	Liquid	OMRON MicroAIR nebulizer	MTT assay on A549 and MRC-5 cell lines	The NEs characteristics nominated it as potential inhalable carriers for docetaxel.	[118]
Docetaxel & Curcumin	PKOE, lauric FA, myristic FA, lecithin, Tween 85^®^, Span 85^®^, & glycerol	To formulate and optimize aerosolized NEs encapsulating docetaxel and curcumin	N/A	Liquid	OMRON MicroAIR nebulizer	N/A	The optimized NE offered desirable physicochemical and aerodynamic properties for inhalation therapy.	[119]
Curcuminoids	Limonene or oleic acid with Tween 80^®^ & ethanol	To prepare nebulized curcuminoid-loaded NEs	N/A	Liquid	Sidestream jet nebulizer	Comet assay on human lymphocytes cells	Both NEs characteristics surpassed the saline-based suspensions of curcuminoid, with no genotoxicity.	[120]
Quercetin	PBE, Tween 80^®^, lecithin & glycerol	To enhance quercetin solubility and cytotoxic selectivity	N/A	Liquid	OMRON MicroAIR nebulizer	MTT assay on A549 and MRC-5 cell lines	Loaded NEs characteristics and release profile were within the pulmonary delivery selection criteria requirements with stable and selective cytotoxic manners.	[121,122]
**SLNs**								
Blank formulation	Lipid mixture (Softisan^®^ & Phospholipon^®^ 90G) & Solutol^®^ HS15 as surfactant.	To evaluate the short-term toxicity of inhaled blank SLNs	N/A	Liquid	A jet-driven aerosol generator system (nebulizer)	MTT assay, neutral red uptake assay on A549 cell line and WST-1 assay using organotypic lung tissue cultures. Short term safety study on female BALB/c mice (age, 8–12 weeks).	This blank SLNs is suitable for pulmonary drug delivery via inhalation. No in vivo record of upregulation in lactate dehydrogenase and inflammation indicators levels	[123]
Erlotinib	Compritol 888 ATO^®,^ Tween 80^®^, poloxamer 407^®^.	To get rapid drug deposition in lungs, with improved drug therapeutic efficiency and less systemic side effects	N/A	Liquid & DP	N/A	MTT assay on A549 cell line	The developed loaded SLNs surpassed the free drug with a cumulative drug release profile and significant higher anticancer activity.	[124]
Epirubicin	Compritol 888 ATO^®,^ lecithin, poloxamer 188^®^.	To overcome major side effects including hematological and cardiac toxicity.	N/A	Liquid	Pari inhalier boy nebulizer	Crystal violet cytotoxicity assay on A549 cell line. Pharmacokinetic study using male SD rats, (250 ± 20 g)	The suitable SLNs characteristics offered a decrease in drug loss, with possible ability to deliver the drug into the deep lung, enhanced cytotoxicity and pharmacokinetics (~ 2 folds)	[125]
Afatinib & paclitaxel	Stearic acid & poloxamer 188^®^.	To explorer the co-delivery outcome of those drugs.	N/A	Liquid & DP	In vitro: Turbospin, a single dose powder inhaler device. In vivo: a dry powder insufflator	Growth-inhibitory curves study on H1975 and PC9/G cell lines. Short-term safety evaluation, pharmacokinetic and tissue distribution using male SD rats, (180–220 g)	The SLNs characteristics offered extremely high retention in the induction port for both drugs and with no interaction between them or the excipients. Pharmacokinetically, SLNs offered 96 h of a two-stage release and high lung concentration, with no signs of other critical organs distribution	[126]
Paclitaxel	Glyceryl-stearate, cholesterol, vitamin E TPGS & sodium taurocholate.	To targeted deliver poor soluble drug	Folate-PEG/chitosan	Liquid	MicroSprayer Aerosolizer IA-1C (endotracheal route)	Cellular uptake on HeLa (CCL-2), M109-HiFR cell lines. Pharmacokinetic study using female (CD-1 and BALB/c mice.	The coated SLNs entered folate receptor (FR)-expressing HeLa and M109-HiFR cells in vitro, and M109 tumors in vivo after pulmonary delivery. The formulation prolonged the pulmonary exposure to paclitaxel up to 6 h and limited systemic distribution.	[42]
Myricetin	Gelucires (G 39/01, 50/13, 44/14) & compritol^®^	To enhance the nutraceutical solubility, stability, and delivery	NA	DP	N/A	MTT assay, and cellular uptake on A549 cell line.	Gelucire-based SLNs were proved to improve the physiochemical properties, release, and anticancer effects of the drug.	[127]
Gefitinib	Lecithin, cholesterol, stearic acid, & PEG2000	To glucosamine targeted	Glucosamine	DP	N/A	MTT assay, and cellular uptake on A549 cell line.	The satisfactory aerosol formulation cellular uptake study clearly demonstrated that functionalization of SLNs with glucosamine promote the accumulation of SLNs within GLUT1 overexpressing cells	[74]
**NLCs**								
Celecoxib	Compritol^®^, miglyol^®^, & sodium taurocholate	Evaluation of anticancer synergetic activity of aerosolized celecoxib-NLCs in combination with IV docetaxel.	N/A	Liquid	Inexpose™ nebulizer	MTT assay on A549 cell line. Tumor size reduction evaluation using nu/nu mice (age, 4–6 weeks).	In vivo study proved the synergetic effects of celecoxib-NLCs inhalation and docetaxel IV.	[128]
Paclitaxel	Stearic acid (or glyceryl monostearate) oleic acid, Tween 80^®^, Tween 20^®^, or Tween 40^®^.	To compare the oral paclitaxel solution with the paclitaxel-NLCs inhaled delivery	N/A	DP	DP insufflator	Intracellular uptake assay in Caco-2 cell line. Organ distribution of loaded NLCs using male Wistar rats, 180–200 g.	Inhaled paclitaxel-NLCs showed excellent local delivery and organ selectivity when accumulated mainly in lung and in compared to pure drug solution oral intake	[129]
Doxorubicin or paclitaxel	Precirol ATO 5^®^, squalene, Tween 80^®^	To provide selective local and targeted inhalation lung delivery.	Synthetic analog LHRH/DSPE-PEG2000 and siRNA	DP	Collision nebulizer connected to nose-only exposure chamber for inhalation	Cellular uptake and the intracellular localization on A549 cell line. Tumor size reduction evaluation using athymic nu/nu mice	The developed inhaled NLCs showed high efficiency and selectivity for tumor-targeted local delivery.	[130]
Paclitaxel	Precirol ATO 5^®^, squalene, Tween 80^®^	To compare the NLC cytotoxicity and selectivity via I.V. and inhalation routes.	LHRH-PEG2000-siRNA	Liquid	Collison nebulizer	MTT assay on A549, H1781, and H3255 cell lines. NLCs organ distribution and tumor size evaluation using nude mice.	Efficient accumulation and retention of the inhaled NLCs in the mice lungs, with no signs of systematic cytotoxicity compared to I.V. route.	[131]
**LPHNs**								
siRNA	PLCGA & DPPC	To downregulate the genes involved in the pathogenesis of LC through the local siRNA delivery	N/A	Liquid	Vibrating mesh nebulizer	MTT assay on A549 and 16HBE14o cell lines	The developed NLCs offered a peculiar triphasic siRNA release lasting for 5 days, with a prolonged inhibition of ENaC protein expression.	[132]
**Niosomes**								
Gemcitabine & cisplatin	Tween 65^®^, Span 60^®^, cholesterol, sodium dodecyl sulfate, glycerol	To develop dual drug inhalable niosomes with efficacy but lower dose, and side effects.	N/A	Liquid	OMRON MicroAIR nebulizer	MTT assay on A549 and MRC5 cell lines	Developed NLCs cytotoxicity reduced against the tested cell lines when compared with free drug.	[133]
Curcumin	Span 80^®^, diethyl ether, with or without cholesterol	To formulate C.-niosomes for effective lung delivery	Cationic niosomes	DP	Nebulizer	MTT assay and cellular uptake on A549 cell line	The cholesterol-containing carriers surpassed the cholesterol-free carriers in terms of antiproliferative effects and a higher endocytosis.	[134]
**Sterosomes**								
Metformin	Cholesterol, stearylamine or myristic acid	To evaluate the safety, tolerability, & pharmacokinetics of inhaled metformin sterosomes	N/A	Liquid	Jet nebulizer	MTT assay on A549 cell line. Clinical study (*n* = 6, 3 males and 3 females age > 18)	The formulated carriers significantly increased the biological half-life area under the curve, and mean residence time of metformin in all healthy volunteers after inhalation.	[135]
**Lipid Nanocapsules**								
Paclitaxel	Captex 8000^®^, Lipoid S75-3^®^, & Solutol^®^ HS 15.	To encapsulate paclitaxel in lipid nanocapsules	N/A	Liquid	Jet, ultrasonic and mesh nebulizers of different brands.	Growth inhibition assay on NCI-H460 human lung cancer cells	LNC dispersions could be made into aerosols by using mesh nebulizers without altering the LNC structure.	[66]

DP: Dry powder; DPPC: Dipalmitoylphosphatidylcholine; DSPE-PEG2000: Glycero-3-phospho-ethanolamine-N-[methoxy(polyethylene glycol)-2000]; FA: Fatty acids; LHRH: Luteinizing hormone-releasing hormone; PBE: Palm-based ester; PKOE: Palm kernel oil esters; PLCGA: Poly(lactic-co-glycolic) acid; SD: Sprague–Dawley; siRNA: Small interfering RNA.

**Table 3:** Summary of clinical trials that have been conducted on the inhalable anticancer drug-loaded lipid-based nanocarriers for the treatment of LC.

**Drug**	Cisplatin	Cisplatin	9-nitrocamptothecin
**NCT** **Number**	N/A *	NCT00102531	N/A **
**Phase**	Phase I	Phase Ib/IIa	Phase I
**Nanocarrier Type**	Liposomes	Liposomes	Liposomes
**Nanocarrier composition**	Dipalmitoyl phosphatidylcholine (DPPC) and Cholesterol	Dipalmitoyl phosphatidylcholine (DPPC) and Cholesterol	Dilauroyl phosphatidylcholine (DLPC)
**Drug dose**	1.5–60 mg/m^2^	24 and 36 mg/m^2^	6.7–26.6 µg/kg/day
**Study duration**	1 to 4 consecutive days in 3-weeks cycles.	The given dose was administered on a 2-weeks cycles.	The given dose was administered for 1 to 8 weeks followed by a 2-weeks rest cycles.
**Delivering device**	Nebulizer	Nebulizer	Nebulizer
**Droplet size**	3.7 ± 1.9 µm	3.7 ± 1.9 µm	1–3 µm
**No. of** **Subjects**	17	19	25
**Type of** **carcinoma**	NSCLC (16) SCLC (1)	High-grade, progressive, or recurrent osteosarcoma in the lungs (secondary LC).	Primary or metastatic LC.
**Subjects’ gender**	F + M	F + M	F + M
**Age, mean (years)**	41.8–70.7, 56.6	13–27, 18 ± 3	33–84, 58.5
**Main adverse events**	Dyspnea, vomiting, nausea, cough, hoarseness, and eosinophilia.	Dyspnea, nausea, cough, and wheezing.	Pharyngitis, fatigue, nausea, vomiting, cough, anemia, neutropenia, anorexia, and skin rash
**Main** **findings**	A significant reversible (in 94% of the subjects) decrease in forced expiratory volume in 1 s (FEV_1_) was observed after one cycle.	Most of the adverse events occurred at the higher given dose (36 mg/m^2^).	A decrease in pulmonary function tests during treatment was noticed.
	No significant change in the diffusing lung capacity for carbon monoxide.	No significant or long-lasting systematic adverse events were noticed.	No hematological toxicities were noticed.
	No dose-limiting toxicity was observed at the maximum delivered dose.	No significant change in the pulmonary function testing parameters.	Inhaled 9NC plasma levels were like those observed after oral ingestion.
	No systematic adverse effects of cisplatin were noticed.	Serum concentrations of inhaled cisplatin were lower than those of intravenous cisplatin.	A dose-dependent increment in both C max and AUC values at the two lower doses; 6.7 and 13.3 µg/kg/day, but not at the highest dose.
	Only 10–15% of the dose reached the site of action.	Systemic cisplatin exposure was minimal.	Partial remissions were observed in 2 patients with uterine cancer, and stabilization occurred in 3 patients with primary lung cancer.
	Very low plasma platinum levels only with the longest repeated inhalations.	No significant difference in cisplatin deposition within the tumors and the surrounding lung tissue.	Higher levels of 9NC were found in the lungs compared to those in the plasma by the end of treatment.
	70% of the subjects showed a stable disease, while 23% of them had a progressive disease.	Two patients had stable disease after 2 cycles, underwent metastasectomy, and remained free from pulmonary recurrence 1 year after initiation of therapy.	The recommended dose for Phase II studies was 13.3 µg/kg/day on a daily 60-min exposure, 5 consecutive days/week for 8 weeks, with a concentration of 9NC of 0.4 mg/mL in the nebulizer.
**Reference**	[30]	[31,32]	[29]

* Phase II clinical trial involving inhaled liposomal cisplatin formulation for the treatment of pulmonary recurrent osteosarcoma (NCT01650090) was completed in 2018 but no data were published yet. ** Totally six clinical trials were conducted furtherly; NCT00492141, NCT00250016, NCT00249990, NCT00250068, NCT00277082 and NCT00250120, where the latter was withdrawn and no published results of the first five trials have been published up to date.

## Data Availability

Data sharing not applicable.
